# Democritus: Homotopy-Localized Causal Discourse Extraction from Language

**DOI:** 10.3390/e28090986

**Published:** 2026-09-03

**Authors:** Sridhar Mahadevan

**Affiliations:** Adobe Research, 345 Park Avenue, San Jose, CA 95110, USA; smahadev@adobe.com

**Keywords:** causal discourse, relative categories, weak equivalence, homotopy localization, non-compositional sketches, natural language processing, causal extraction

## Abstract

Natural-language documents contain many causal claims, but those claims are unstable under paraphrase, granularity shifts, and contextual drift. A collection may express one mechanism in many surface forms, while neighboring studies may agree locally yet fail to glue globally because relation families, polarities, temporal scopes, or regimes differ. This paper studies that post-extraction problem through Democritus, an implemented system for extracting, normalizing, localizing, and diagnosing causal discourse. We do not claim to identify ground-truth causal structure from text alone. Instead, we formulate the post-extraction layer as a homotopical repair problem for a non-compositional causal-discourse sketch. A normalization functor induces a class of weak equivalences on textual mentions; we prove that these data form a relative category with two-out-of-three and that normalization factors through its localization. Strict repair requires a corpus diagram to satisfy the sketch exactly, whereas homotopy repair asks for a weakly equivalent diagram that factors coherently after localization. We also define the conditions under which grounded discourse localization maps functorially into the observational-equivalence spaces of causal models studied by higher algebraic K-theory. The implementation approximates these formal constructions through normalized claim classes, an observed compatibility complex, regime-sensitive gluing, and provenance-preserving database artifacts. On a frozen 404-passage AltLex/UniCausal comparison, a restricted verbatim-span Democritus extractor attains 57.1% causal-classification F1 and 39.1% macro span F1, compared with 59.2% and 43.6% for the public UniCausal baseline. This similar classification F1 reflects a clear precision–recall tradeoff: Democritus achieves higher recall (80.9% versus 68.7%) but lower precision (44.1% versus 52.0%). Case studies on Emperor-penguin climate discourse, Mediterranean-diet studies, red-wine cardiovascular studies, and rising-ocean-temperature corpora expose both stable repeated mechanisms and cases where local causal claims cannot be assembled into one coherent corpus-level account.

## 1. Introduction

This paper studies a fundamental problem in extracting causal discourse from natural-language documents: how to consolidate expressed causal claims without either overcounting paraphrases or erasing scientifically meaningful distinctions. Scientific articles, policy reports, review essays, and news analyses all contain causal discourse. They describe mechanisms, risks, interventions, and explanatory pathways that researchers and decision-makers read long before those claims have been formalized as structural causal models. Yet this discourse is unstable. The same mechanism may be expressed in several phrasings, at several granularities, and under several regimes. Conversely, two sentences that sound similar may differ in polarity, temporal scope, intervention type, or context. A useful system must therefore do more than extract isolated cause–effect records. It must decide which mentions should be identified, which should remain separate, and where local agreements fail to assemble into a coherent global account.

**Figure 1:**
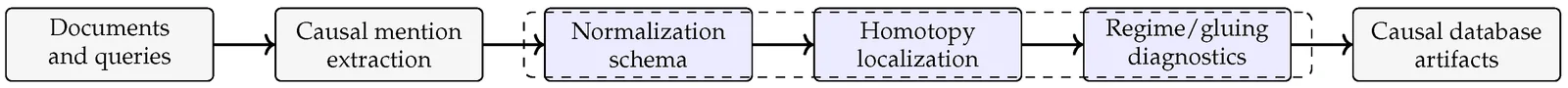
Illustration of the Democritus pipeline. The upstream system extracts candidate causal mentions from documents; the post-extraction layer normalizes those mentions, localizes paraphrastic variants, diagnoses regime-sensitive gluing failures, and preserves the resulting evidence in causal database artifacts. The dashed box marks the normalization, localization, and gluing stages that are the focus of this paper.

**Figure 2:**
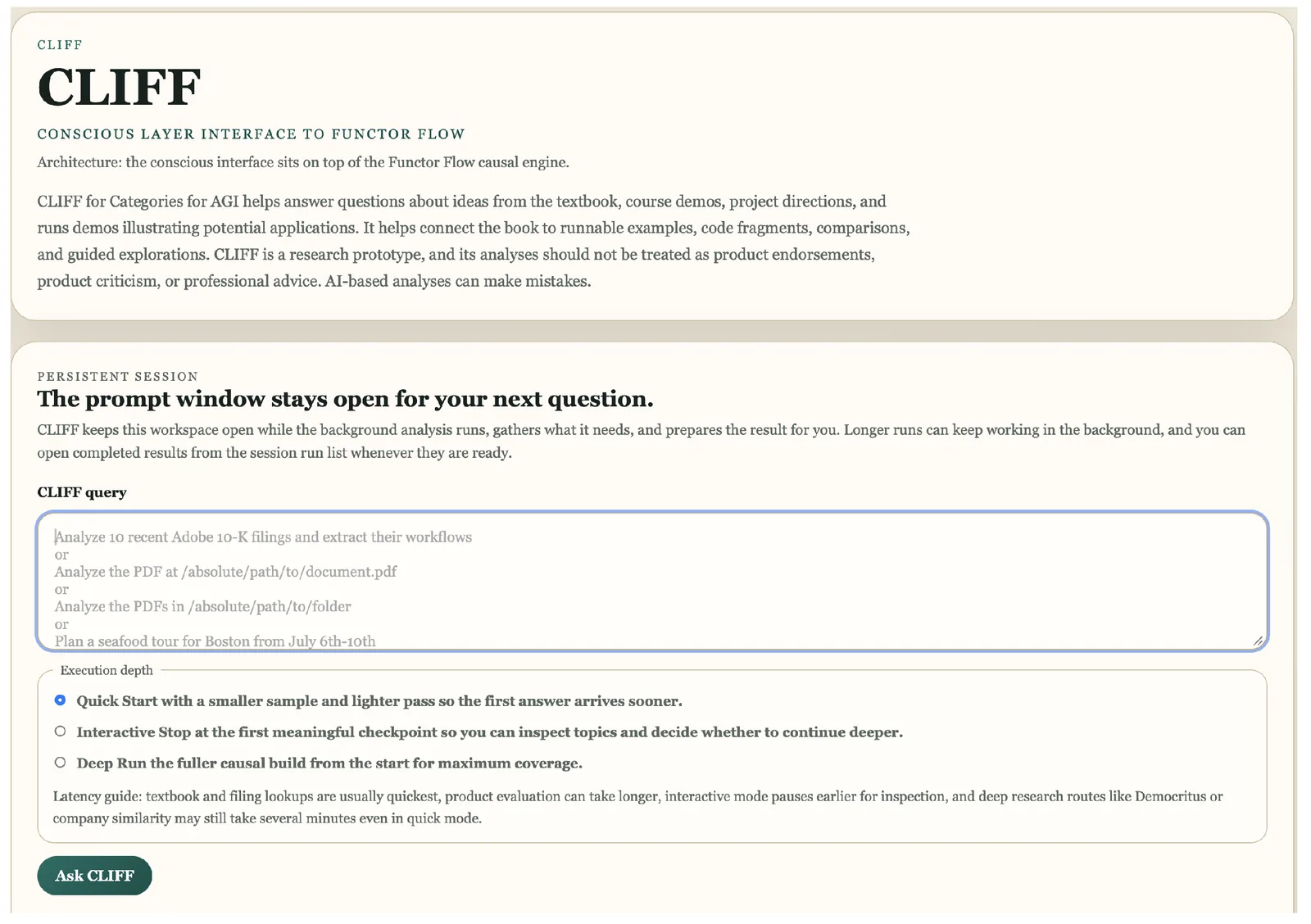
Cliff is the user-facing route layer through which Democritus analyses are launched. User queries are routed to document acquisition, causal extraction, localization, and artifact generation workflows.

We describe this problem through Democritus, an implemented system for extracting and organizing causal discourse from documents [1]. Earlier versions of Democritus built large causal atlases from input texts and stored large families of local causal models in a compact causal SQL representation called Csql [2]. The present paper focuses on a later layer of the system: homotopy localization of extracted causal mentions, regime-sensitive gluing diagnostics, and archived artifacts that make the resulting equivalence classes and failures inspectable. Figure 1 locates that layer inside the broader document-to-artifact pipeline. The current user-facing interface is Cliff, illustrated in Figure 2, which routes natural-language requests into Democritus workflows and returns bounded diagnostic artifacts.

We do not claim that Democritus identifies ground-truth causality from language alone. Taken as a claim about identifiability, that would be false: text does not supply the interventions, experimental controls, or statistical assumptions required by modern causal inference [3,4,5,6,7,8,9]. Our claim is more modest and more operational. Given a corpus containing causal discourse, the system extracts putative causal mentions, normalizes them into structured claims, localizes away paraphrastic variation, and reports where claims glue cleanly, remain regime-sensitive, or cannot be merged into one coherent corpus-level account.

The conceptual language used here is categorical, but the empirical target is concrete. Homotopy localization is used as a computational account of when textual mentions should be treated as equivalent after normalization. A causal-discourse learning sketch specifies which normalization paths, overlap views, and corpus-level assemblies should be composed. Pullback- and pushout-style operations describe how document-local atlases are aligned and merged through canonical interfaces. An observed compatibility complex reports whether families of paraphrases close up coherently under the multiway comparisons actually performed. These constructions are not presented as a replacement for causal discovery from data. They make a particular post-extraction layer more stable, explicit, and auditable.

This distinction also fixes the evidential claim of the paper. The experiments do not show that category theory improves sentence-level extraction accuracy over UniCausal or other causal-relation extractors. They show that, once candidate causal mentions have been extracted, the categorical formulation supplies a disciplined interface between normalization, equivalence, gluing, and obstruction. Without that interface, the implementation could still cluster normalized strings, but it would lose a principled separation between admissible paraphrase collapse and incompatible regime shift; it would also lose the explicit pullback/pushout audit trail used to distinguish shared cross-study mechanisms from document-local claims. The mathematical contribution is therefore a specification of the post-extraction invariants and failure modes, while the engineering contribution is the approximate, inspectable realization of those invariants in Csql artifacts and case-study diagnostics.

The contributions of the paper are fivefold.

1. We prove that the normalization-induced weak equivalences make the category of causal mentions into a relative category satisfying two-out-of-three, and that normalization factors through the corresponding localization.
2. Building on our recent work on Learning in Infinitesimal Non-Compositional Sketches (Lincs) [10], we cast post-extraction as homotopical repair of a causal-discourse learning sketch, separating strict factorization from factorization after weak equivalence.
3. We define a relative-functor criterion under which localized causal discourse maps into observational-equivalence classes of formal causal models, connecting the present construction to the classifying-space perspective of higher algebraic K-theory [11].
4. We give an operational normalization schema and describe its realization in Democritus and Cliff through Csql claim classes, regime gluing, observed compatibility complexes, topic partitions, archived runs, and reproducible artifacts.
5. We report a frozen comparison with the public UniCausal causal-relation extractor and selected empirical results from saved runs. The benchmark exposes a high-recall, lower-precision extraction regime, while the case studies show stable localized families, auditable pullback and pushout witnesses, and fragmented covers that resist a single coherent global merge.

The rest of the paper is organized as follows. Section 2 gives a concrete running example before introducing the formal language. Section 3 describes the scientific challenges in building Democritus. Section 4 defines normalization-induced weak equivalence, localization, and homotopical sketch repair. Section 5 connects localized discourse to causal-model equivalence and gives a condensed account of causal grounding. Section 6 summarizes the operational workflow supporting the implementation. Section 7 summarizes the current Democritus/Cliff pipeline. Section 8 presents selected case studies. Section 9 summarizes related work, and Section 10 discusses limitations and future directions.

## 2. Running Example: From Text to Localized Causal Claims

We use Sarah Kaplan’s 9 April 2026 Washington Post article “Emperor penguins have just been declared endangered” (https://www.washingtonpost.com/climate-environment/2026/04/09/emperor-penguin-endangered-antarctic-fur-seal/ (accessed on 10 April 2026)) as the running example. The goal is not to reproduce or summarize the article, but to expose the causal mentions that the system extracts and the normalization decisions that determine whether those mentions are merged.

The simplified passage below contains the kind of language that appears in the run: warming ocean conditions affect krill distribution; reduced prey availability affects penguin and seal foraging; and sea-ice loss damages emperor-penguin breeding habitat. Democritus first extracts raw causal mentions, illustrated in Table 1, then maps them into a normalized schema.

Table 2 illustrates why homotopy localization cannot be ordinary lexical clustering. Mentions $m_{1}$ and $m_{2}$ are closely related, but they are not identical normalized claims. One describes a displacement of krill into deeper waters; the other describes a reduction in surface availability. They may form a coherent two-step mechanism, but collapsing them into one claim would erase the intermediate ecological state. By contrast, if another sentence said “higher sea-surface temperatures push krill into deeper water”, it would be weakly equivalent to $m_{1}$ because the normalized cause, effect, relation family, polarity, temporal scope, and regime would match.

The resulting output is therefore not a flat list of isolated cause–effect records. It is a small localized causal neighborhood: paraphrases of the temperature–krill-depth claim are aggregated into one class; the surface-krill, food-availability, sea-ice, habitat, and mortality claims remain distinct but linked; and polarity or regime conflicts are reported as sensitive or obstructed rather than silently averaged away. Table 3 summarizes the corresponding merge, link, and non-merge decisions. This is the operational behavior that the formal language below is designed to capture.

## 3. Scientific Challenges in Building Democritus

Large language models can readily generate causal narratives, but extracting stable causal discourse from language remains difficult. The same causal statement may appear in many paraphrastic forms, while neighboring studies may agree locally yet fail to glue globally because relation families or polarities change across regimes. A naive extraction system therefore tends to overcount evidence when paraphrases are left separate, and to overglue evidence when superficially similar statements are collapsed too aggressively. These are not merely presentation issues. They change which claims appear credible, which mechanisms seem to recur across studies, and whether a corpus can be synthesized into a coherent causal account.

Given a query or a local collection of PDFs, Democritus retrieves documents, expands a topic cover, extracts large numbers of causal mentions, constructs thousands of local causal models (LCMs), aggregates them into a causal database, and returns interactive artifacts including executive summaries, topic atlases, manifold views, local causal graph galleries, and corpus-level diagnostics. In recent versions of the system, the central practical bottleneck has not been local graph generation itself, but the instability of causal extraction under paraphrase and contextual drift. The present paper should therefore be read as a study of a specific computational problem inside a working system: how to organize causal discourse so that equivalence, disagreement, and gluing failures become visible rather than being flattened away.

Three challenges recur throughout the saved runs. First, *paraphrase inflation*: one mechanism may be counted many times because the wording changes from sentence to sentence or document to document. Second, *over-localization*: a system may merge claims that share surface vocabulary but differ in sign, mechanism, intervention type, or regime. Third, *cover drift*: the retrieved corpus may remain topically related while decomposing into several local scientific neighborhoods that should not be forced into one global backbone. The running example above illustrates the first two issues inside a single article; the ocean-temperature case study below illustrates the third issue at corpus scale.

The paper is intentionally experimental: it uses categorical language to organize the computational failure modes that arise when causal discourse is extracted from language. Here we focus on what happens in practice when thousands of overlapping causal mentions must be normalized, localized, compared, and preserved in auditable artifacts. This framing clarifies the novelty of the present work. The contribution is not another sentence-level causal relation extractor, nor another claim that a language model “understands causality.” It is a systems-and-theory account of what happens after extraction.

## 4. Homotopy Localization of Causal Mentions

In Democritus, *homotopy localization* means that causal extraction is carried out not at the level of raw textual mentions, but at the level of equivalence classes of mentions that preserve the same normalized causal content. A corpus may express one mechanism through many paraphrases, clause reorderings, or local discourse variants; if those are left separate, evidence fragments and support are overcounted. At the same time, linguistically nearby statements can differ in polarity, temporal scope, intervention type, or regime, so collapsing them too aggressively destroys scientifically important distinctions. Homotopy localization is the computational step that quotients away causally inessential variation while preserving the distinctions that matter for gluing, obstruction, and downstream causal interpretation.

We begin with the syntax side. Let $S_{\mathrm{causal}}$ denote a category of causal mentions, whose objects are sentence fragments, passages, or short discourse neighborhoods expressing putative causal content. Morphisms in $S_{\mathrm{causal}}$ represent semantics-preserving rewrites, paraphrases, normalization steps, or discourse-level reformulations. On the semantic side, let $C_{\mathrm{causal}}$ denote a category of normalized causal claims. A normalization pipeline maps each mention to a structured causal object

c=(X,Y,r,s,τ,κ,q),

where *X* is a cause expression, *Y* an effect expression, *r* a normalized relation family, *s* a polarity, τ a temporal scope, κ a regime or context label, and *q* auxiliary qualifiers such as hedging, confidence, or provenance. We write

$$E:S_{\mathrm{causal}}\to C_{\mathrm{causal}}$$

for the resulting causal extraction functor.

Operationally, the normalization pipeline has two parts. First, an LLM-assisted extraction route proposes candidate cause–effect mentions together with textual provenance. Second, Democritus maps those candidates into a controlled schema: relation families are canonicalized, polarity is made explicit, temporal and regime fields are recorded when available, and qualifiers preserve hedging, source identity, and confidence. The most common residual failure modes are polarity confusion in hedged prose, ambiguous or missing regime labels, clause-tail drift in long mechanism chains, granularity mismatch between broad and specific effects, and retrieval-cover drift that mixes neighboring scientific subdomains. Table 4 summarizes the fields that determine whether two mentions are eligible to become weakly equivalent in the implemented system.

### 4.1. What the Categorical Layer Adds Operationally

It is useful to separate the normalization machinery, which produces structured claim records, from the categorical layer, which specifies how those records may be compared and assembled. A system without the categorical layer could still canonicalize strings, group similar claims, and count support. What it would lack is an explicit account of which identifications are admissible, which diagrams fail to compose after those identifications, and which merge operations preserve provenance over a shared interface.

This functor defines the notion of weak equivalence used throughout the paper. A morphism f:u→v in $S_{\mathrm{causal}}$ is a weak equivalence when E(f) is an isomorphism in $C_{\mathrm{causal}}$. Write $W_{E}=\left(\right|S_{\mathrm{causal}}\left|,\Sigma_{E}\right)$, with source, target, identities, and composition inherited from $S_{\mathrm{causal}}$, where $|S_{\mathrm{causal}}|$ contains all the objects of $S_{\mathrm{causal}}$, and 

$$\Sigma_{E}=\left\{f\in \mathrm{Mor}\left(S_{\mathrm{causal}}\right)|E\left(f\right)\;\mathrm{is}\;\mathrm{an}\;\mathrm{isomorphism}\right\}.$$

Intuitively, weak equivalences are rewrites that preserve normalized causal content. They are not mere lexical similarities. Two mentions may sound close yet fail to be weakly equivalent if they differ in sign, intervention type, temporality, or regime. This is why the extraction functor is essential: it prevents homotopy from collapsing distinctions that are causally meaningful.

**Proposition** **1** (Normalization-induced relative category)**.** *Let $E:S_{\mathrm{causal}}\to C_{\mathrm{causal}}$ be a functor and let $W_{E}$ be defined as above. Then $W_{E}$ is a wide subcategory of $S_{\mathrm{causal}}$ and satisfies the two-out-of-three property. Consequently $\left(S_{\mathrm{causal}},W_{E}\right)$ is a relative category whose weak equivalences satisfy two-out-of-three.*

**Proof.**  For every object *u*, $E\left({\mathrm{id}}_{u}\right)={\mathrm{id}}_{E(u)}$ is an isomorphism, so every identity lies in $\Sigma_{E}$. If *f* and *g* are composable elements of $\Sigma_{E}$, then E(gf)=E(g)E(f) is an isomorphism, proving closure under composition. Together with the inclusion of every object by definition, these facts make $W_{E}$ a wide subcategory. Finally, for composable *f* and *g*, if any two of E(f), E(g), and E(gf)=E(g)E(f) are isomorphisms, then so is the third. Hence $W_{E}$ satisfies two-out-of-three. □

Relative categories provide the minimal setting for studying objects up to a specified class of weak equivalences [12]. Under the usual smallness or universe conventions, let

$$Q:S_{\mathrm{causal}}⟶S_{\mathrm{causal}}\left[\Sigma_{E}^{-1}\right]$$

be the Gabriel–Zisman localization.

**Proposition** **2** (Normalization descends through localization)**.** *There is a functor*

$$\bar{E}:S_{\mathrm{causal}}\left[\Sigma_{E}^{-1}\right]⟶C_{\mathrm{causal}}$$

*such that $\bar{E}Q=E$, unique up to the canonical uniqueness of the localization factorization.*

**Proof.**  By definition, *E* sends every morphism in $\Sigma_{E}=\mathrm{Mor}\left(W_{E}\right)$ to an isomorphism. The claim is therefore the universal property of localization. □

Thus the relevant homotopy category is

$${\mathrm{Ho}}_{E}\left(S_{\mathrm{causal}}\right)\;:=\;S_{\mathrm{causal}}\left[\Sigma_{E}^{-1}\right].$$

Mentions connected by a zig-zag of weak equivalences become isomorphic, although the objects of the localization are not literally set-theoretic equivalence classes. This is the mathematical form of the operational instruction “merge paraphrases that express the same causal claim.” The canonical claim classes stored in the Csql bundle are a computational quotient approximating this localization: evidence is aggregated at the level of normalized causal content rather than unstable wording.

### 4.2. Homotopical Repair of a Causal-Discourse Learning Sketch

Localization identifies admissible variation, but it does not by itself say which local claims should compose. For that purpose, define a causal-discourse learning sketch [10]:

$$S_{\mathrm{Dem}}=\left(S,D,L,K\right).$$

The graph *S* contains typed generators for mentions, normalized claims, documents, regimes, overlaps, and proposed corpus-level assemblies. The path equations D require alternative normalization or refinement routes to agree. The distinguished cones L encode document- and regime-local views over their shared interfaces, while the distinguished cocones K encode proposed provenance-preserving assemblies of compatible claims. Let J=Path(S) and let

$$q_{D}:J⟶J/{∼}_{D}$$

be the quotient by the path equations.

A corpus realization is a diagram

$$D:J⟶S_{\mathrm{causal}}$$

together with the selected cone and cocone data. Strict compositionality requires a factorization $D=\bar{D}q_{D}$ that also realizes the specified universal constraints. This is too rigid when two extraction paths differ only by a normalization-preserving rewrite. We therefore define homotopical compositionality by asking for a functor

$$\bar{D}:J/{∼}_{D}⟶{\mathrm{Ho}}_{E}\left(S_{\mathrm{causal}}\right)$$

and a natural isomorphism

$$QD≅\bar{D}q_{D},$$

with the images of the distinguished cones and cocones satisfying their localized requirements. A *homotopy repair* replaces or augments the observed diagram by weakly equivalent data for which this localized factorization is inhabited. The primitive obstruction is the failure of this factorization problem, not an arithmetic distance between two embeddings.

This construction is the homotopical base-repair layer associated with Learning in Infinitesimal Non-Compositional Sketches (Lincs) [10]. It does not yet constitute infinitesimal non-compositionality: the present Democritus implementation supplies no tangent functor *T* and does not compute the tangent factorization problem for TD. A future tangent model could test whether a repaired discourse diagram remains compositional under perturbations of extraction, normalization, or regime assignment. The current contribution is the prior global question of compositionality up to the declared weak equivalences.

### 4.3. Observed Compatibility and Coherent Completion

To expose higher-order compatibility without presupposing it, let $K_{c}^{\mathrm{obs}}$ be the observed compatibility simplicial complex associated with a normalized claim *c*. Its vertices are extracted mentions eligible to represent *c*; its edges are pairwise compatibility decisions actually recorded by the system; and an *n*-simplex is included only when the corresponding (n+1)-way comparison has been certified as jointly compatible. Face closure records the requirement that a verified multiway comparison restricts it to verified lower-order comparisons. A missing candidate filler can, therefore, record a genuine absence or failure of observed coherence.

This observed object must be distinguished from the nerve of a paraphrase groupoid. If $G_{c}$ is a genuine groupoid of coherently invertible paraphrase maps, then its nerve $N(G_{c})$ is a Kan complex and every horn has a filler. It is therefore an idealized coherently completed object, not an object in which missing fillers diagnose failure. The implemented diagnostic concerns $K_{c}^{\mathrm{obs}}$ and its possible completion toward such a coherent object.

This distinction between paraphrase and regime is essential for the present paper. One article may say that elevated sea surface temperatures affect habitat suitability for cold-adapted species, while another says they reduce it. Linguistically, these are close, but the polarity conflict means they should not become equivalent in ${\mathrm{Ho}}_{E}\left(S_{\mathrm{causal}}\right)$. The practical question is therefore not merely which mentions are similar, but which mentions are admissibly equivalent once relation, family, polarity, temporal scope, and regime are respected. That is the sense in which homotopy localization is stronger than surface paraphrase collapse: it stabilizes causal discourse without erasing scientifically important distinctions. With that stabilization layer in place, we can then ask how localized discourse objects are grounded in formal causal models.

## 5. A Categorical Bridge from Discourse to Models

### 5.1. Formal Definitions and Implemented Analogues

Before introducing the categorical bridge, it is important to state how literally the formal language should be read. The normalization-induced relative category, its localization factorization, the homotopical sketch-factorization problem, Proposition 3, and the category-of-elements formula in Theorem 1 are ordinary mathematical constructions. Their hypotheses must nevertheless be checked for any concrete implementation. The Csql database stores canonical claim classes and equivalence links rather than a theorem-prover-certified localization; the red-wine pullback and pushout experiments are database alignment and merge operations over a canonicalized interface rather than formal limit computations; and $K_{\left[c\right]}^{\mathrm{obs}}$ records finite observed compatibility rather than a completed homotopy type. Table 5 states the operational payoff of these constructions. The formal constructions specify invariants and universal properties the system approximates; the saved artifacts expose the corresponding operational decisions for audit. Consequently, the theoretical results support the practical conclusions only at the level of specification and diagnostic interpretation. They do not by themselves validate extraction accuracy, merge correctness, or causal truth.

### 5.2. Discourse Space and Model Space

The separation between a space of linguistic causal discourse and a space of formal causal models parallels familiar distinctions in categorical semantics of language. In Lambek-style and related compositional accounts, syntax lives in one structured category while semantics is assigned in another, and enriched-meaning constructions explain why some expressions require a lifted semantic space only when composition makes that necessary [13,14]. Democritus admits an analogous reading.

Let $M_{\mathrm{disc}}$ denote a category of causal discourse objects: document neighborhoods, discourse motifs, and extracted claim families whose content is expressed in natural language and whose intervention semantics is only implicit. Let $M_{\mathrm{model}}$ denote a category of formal causal models, including structural causal models and related categorical causal-model objects, in which interventions, transport, and identification are defined explicitly.

On this view, Democritus v1 mostly lives in $M_{\mathrm{disc}}$: it samples and organizes causal discourse, builds neighborhoods, and surfaces recurrent mechanisms. The missing step is *causal grounding*: a principled passage from discourse neighborhoods to formal causal objects on which operations such as adjustment or transport can be carried out. That passage is what motivates the adjoint formulation below.

### 5.3. From Discourse Equivalence to Causal-Model Equivalence

The localization above acts on discourse representations. It should not be confused with observational equivalence of formal causal models. Earlier work studied the latter through cPROP causal functors, transformations generated by covered-edge moves, nerves, classifying spaces, and higher algebraic K-theory [11]. The two constructions occupy adjacent semantic levels: Democritus organizes unstable linguistic evidence, whereas causal-model equivalence identifies formal models with the same observational content.

To state their relationship, let $S_{\mathrm{disc}}^{g}$ be a category of *grounded* causal-discourse objects for which an explicit formal causal interpretation has been supplied. Equip it with the wide subcategory $W_{\mathrm{disc}}$ of normalization-induced weak equivalences, and write $\Sigma_{\mathrm{disc}}=\mathrm{Mor}\left(W_{\mathrm{disc}}\right)$. Let $M_{\mathrm{causal}}$ be a selected category of formal causal models, equipped with a wide subcategory $W_{\mathrm{obs}}$ of observational equivalences, with $\Sigma_{\mathrm{obs}}=\mathrm{Mor}\left(W_{\mathrm{obs}}\right)$. A grounding functor

$$\Phi :\left(S_{\mathrm{disc}}^{g},W_{\mathrm{disc}}\right)⟶\left(M_{\mathrm{causal}},W_{\mathrm{obs}}\right)$$

is *relative* when

$$f\in \Sigma_{\mathrm{disc}}\;⟹\;\Phi \left(f\right)\in \Sigma_{\mathrm{obs}}.$$

This condition says that changing the textual representative without changing its normalized causal content cannot move the grounded object to a different observational-equivalence class.

**Proposition** **3** (Grounding localized discourse)**.** *If* Φ *is relative, then it induces a functor*

$$\bar{\Phi }:S_{\mathrm{disc}}^{g}\left[\Sigma_{\mathrm{disc}}^{-1}\right]⟶M_{\mathrm{causal}}\left[\Sigma_{\mathrm{obs}}^{-1}\right]$$

*for which the following square commutes up to the canonical localization isomorphism:*

$$\begin{array}{c} S_{\mathrm{disc}}^{g}\overset{\Phi }{⟶}M_{\mathrm{causal}}\\ Q_{\mathrm{disc}}↓\;\;↓Q_{\mathrm{obs}}\;\;\\ \;\;\;S_{\mathrm{disc}}^{g}\left[\Sigma_{\mathrm{disc}}^{-1}\right]\underset{\bar{\Phi }}{\to }M_{\mathrm{causal}}\left[\Sigma_{\mathrm{obs}}^{-1}\right]. \end{array}$$

**Proof.**  The composite $Q_{\mathrm{obs}}\Phi$ sends every morphism in $\Sigma_{\mathrm{disc}}$ to an isomorphism because Φ is relative and $Q_{\mathrm{obs}}$ inverts the morphisms in $\Sigma_{\mathrm{obs}}$. The universal property of $Q_{\mathrm{disc}}$ therefore supplies $\bar{\Phi }$, uniquely up to the canonical localization isomorphism. □

Applying the nerve and geometric realization gives a map between the corresponding classifying spaces. This establishes a precise route from homotopy-localized discourse to the causal-equivalence spaces studied in higher algebraic K-theory. It does *not* imply that the current system computes algebraic K-theory. An induced map of K-theory spaces would require additional exact, Waldhausen, or symmetric-monoidal structure and preservation properties that are not established here.

The restriction to $S_{\mathrm{disc}}^{g}$ is essential. An extracted sentence does not determine a formal SCM merely by being normalized. Constructing Φ requires variables, graph or mechanism structure, and explicit causal assumptions. In the present implementation, canonical claim classes are candidates for such grounding, not automatically certified causal models.

### 5.4. The Discourse–Model Adjunction

At the level of specification, suppose that formalization and explanation form an adjunction

$$F⊣G:M_{\mathrm{disc}}⇄M_{\mathrm{model}},$$

where *F* formalizes discourse objects into causal models and *G* maps causal models back to canonical explanatory discourse. The adjunction gives a natural bijection

$${\mathrm{Hom}}_{M_{\mathrm{model}}}\left(F\left(d\right),m\right)\;≅\;{\mathrm{Hom}}_{M_{\mathrm{disc}}}\left(d,G\left(m\right)\right).$$

**Figure 3:**
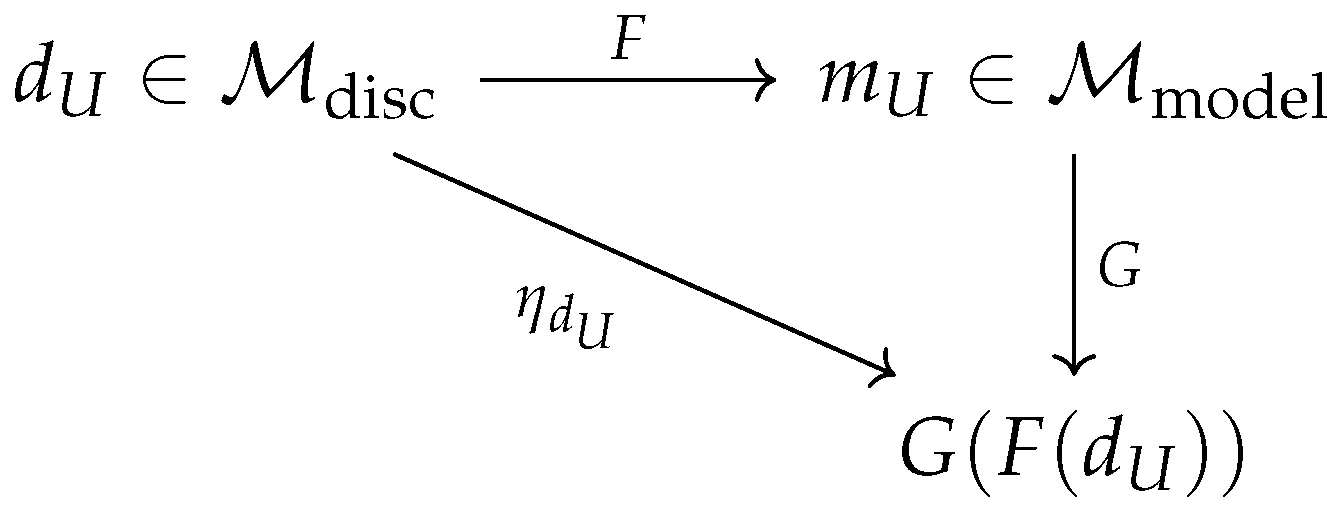
The unit of a candidate discourse–model adjunction. If the formalization and explanation functors form an adjunction, formalizing a discourse neighborhood and then explaining the resulting model back into language should preserve causal content up to normalization and explicit assumptions.

If such an adjunction exists, fitting the formalization of a discourse neighborhood into a model is equivalent to mapping the original neighborhood into the canonical explanation of that model. Its unit η:Id⇒GF and counit ϵ:FG⇒Id become round-trip consistency contracts: formalize-then-explain should preserve discourse up to normalization, while explain-then-formalize should return to the original model up to conservative approximation. The live system does not establish this general adjunction. The next subsection constructs a precise instance, with explicit hypotheses, on a presheaf category of typed discourse generators. Figure 3 depicts the unit and its formalize–explain round trip.

### 5.5. A Universal Property for Causal Grounding

To make this bridge precise, let D be a small category of typed causal discourse generators: atomic discourse templates, typed motifs such as back-door, front-door, instrument, collider, selection, and transport fragments, together with refinement or embedding maps between them. Let E be a cocomplete category of causal models, and let

A:D→E

interpret each discourse generator as a primitive model fragment.

Define the discourse-observation functor

$$R:E\to {\mathrm{Set}}^{D^{op}},\;R\left(E\right)\left(d\right):={\mathrm{Hom}}_{E}\left(A\left(d\right),E\right).$$

Thus, R(E) records which discourse motifs are realized inside the model *E*. The left adjoint to *R* is obtained by gluing model fragments according to a presheaf of discourse constraints.

**Theorem** **1** (Discourse Realization by Colimit)**.** *Let D be a small category, let E be cocomplete, and let A:D→E be any functor. For each presheaf $P\in {\mathrm{Set}}^{D^{op}}$, define*

$$L\left(P\right):=\mathrm{colim}\left(\int_{D}P\overset{\pi_{P}}{\to }D\overset{A}{\to }E\right),$$

*where $\int_{D}P$ is the category of elements of P. Then L is left adjoint to R, so there is a natural bijection*

$$\mathrm{Nat}\left(P,R\left(E\right)\right)≅{\mathrm{Hom}}_{E}\left(L\left(P\right),E\right)$$

*for every presheaf P and model object E in E. If moreover A is dense on the full subcategory of E generated under colimits by its image, then every motif-generated model is canonically recovered from its discourse observations.*

**Proof.**  This is the standard category-of-elements formula for the left Kan extension of *A* along the Yoneda embedding. The colimit defining L(P) glues together copies of the primitive fragments A(d) indexed by generalized elements of *P*, and the universal property of that colimit yields the stated natural bijection. Density of *A* is then the usual density-theorem criterion guaranteeing that the induced nerve functor is fully faithful on the motif-generated subcategory. □

The theorem is useful because it identifies causal grounding with a universal property rather than with a specific implementation detail. A discourse presheaf *P* represents local typed evidence; L(P) is the canonical causal model assembled from that evidence; and *R* extracts the family of discourse views supported by any model. In particular, the induced monad

T:=RL

acts on discourse presheaves as a *causal grounding closure*: realize the discourse as a model and then re-express the supported views in discourse space. This is the categorical form of the enrichment step that Democritus approximates computationally.

When E is taken to be a category of local structural causal models [3] or another categorical model class with suitable colimits, the same theorem says that a small generating family of typed motifs can be dense enough to recover an experimentally relevant class of causal models from motif observations alone. That is the precise sense in which the discourse–model adjunction is more than metaphor.

### 5.6. Worked Grounding Examples

The abstraction above becomes concrete in familiar identification settings. The examples in this subsection are typed illustrations conditional on a correct grounding map; they are not claims that the current text pipeline has certified the required graph or identification assumptions. Consider a discourse neighborhood $d_{U}$ containing the claims: smoking increases lung-cancer risk; age affects smoking; age affects lung cancer; smoking is correlated with age; and controlling for age still leaves a smoking effect. A supplied formalization functor can map this neighborhood to a model with variables X= Smoking, Y= LungCancer, and Z= Age together with graph

Z→X→Y,Z→Y.

In model space the relevant operation is back-door adjustment:

$$P\left(Y|do\left(X=x\right)\right)=\sum_{z}P\left(Y|X=x,Z=z\right)P\left(Z=z\right).$$

The explanation functor *G* then renders this model-level derivation back into canonical discourse: age is a confounder, the back-door path is blocked by conditioning on age, and the causal effect is therefore identifiable under the stated assumptions. The point is not that the original text contained the formula verbatim; it is that the discourse neighborhood carries enough structured content for a formal model to make the identification criterion explicit.

Front-door identification gives an even clearer local-to-global picture. Suppose a discourse neighborhood asserts that smoking causes tar deposits, tar causes lung cancer, smoking has no direct effect on lung cancer except through tar, and there are unobserved confounders between smoking and lung cancer. A chosen formalization produces variables X= Smoking, Z= Tar, and Y= LungCancer with graph X→Z→Y and latent confounding between *X* and *Y*. Back-door adjustment fails, but front-door transport succeeds through local sections:

P(z∣do(x))=P(z∣x)

and

$$P\left(y|do\left(z\right)\right)=\sum_{x^{\prime }}P\left(y|z,x^{\prime }\right)P\left(x^{\prime }\right).$$

Gluing these yields

$$P\left(y|do\left(x\right)\right)=\sum_{z}P\left(z|x\right)\sum_{x^{\prime }}P\left(y|z,x^{\prime }\right)P\left(x^{\prime }\right).$$

This is naturally sheaf-like: each conditional distribution is a local section over a variable patch, and identification is a gluing operation, producing a global interventional section from compatible local ones. In Democritus, regime gluing is a discourse-level analogue of this logic. Not every family of local claims descends to a global statement, and obstruction is diagnostically meaningful.

### 5.7. Simplicial Diagnostics for Localized Claim Families

Homotopy localization explains when surface forms should be identified. In the implemented system, the next question is not the full homology of a paraphrase complex, but whether a localized family closes up under the multiway comparisons that Democritus actually performs. For a normalized claim class [c], the finite object $K_{\left[c\right]}^{\mathrm{obs}}$ is the observed compatibility complex defined in Section 4.2: vertices are surface realizations, edges record accepted pairwise compatibility after normalization, and higher simplices are recorded only when the corresponding multiway agreement is explicitly verified.

The diagnostics are consequently finite and auditable. If the observed family is well connected and its tested multiway comparisons close under the required faces, Democritus reports a *coherent* localized class. If some local compatibilities exist but tested multiway comparisons fail or remain uncertified, the system reports a *partially glued* family, meaning that nearby formulations align only locally or only under some regime interpretation. If the family breaks into separate compatibility components, it is reported as *disconnected*, which in practice often signals clause-tail segmentation, latent mechanism splitting, or a genuine shift in discourse regime.

This viewpoint clarifies why the present implementation reports coherent, partially glued, and disconnected families rather than only a single similarity score. The current simplicial diagnostics are intentionally lightweight: they ask not merely whether two claims are close, but whether an entire observed family closes coherently under tested comparisons. They do not compute the nerve of a completed groupoid, a fibrant replacement, homology, or another full topological invariant.

## 6. Operational Workflow Behind Democritus

The previous sections explained why localization, gluing, and obstruction are useful concepts for causal discourse extracted from text. This section states how those ideas appear operationally in the implemented workflow. The input is a set of document-local causal artifacts: extracted causal statements, local causal models, topic neighborhoods, relation labels, and provenance links. The output is a bounded synthesis object: localized claim classes, regime-sensitive gluing labels, manifold or topic views, and causal database artifacts that can be inspected after the run.

Table 6 summarizes the three workflow roles used in the experiments: geometric organization of extracted claims, consistency checking over local views, and aggregation into auditable corpus-level artifacts. We refer to these roles as Geometric Transformers (GT), Diagrammatic Backpropagation (DB), and Kan Extension Transformers (KET) in the implementation stack.

Read in this operational order, the pipeline is straightforward. GT first supplies neighborhoods: it decides which extracted claims, local causal models, and topics are close enough to compare. Homotopy localization then uses the normalized claim schema to identify paraphrastic variants inside those neighborhoods. DB supplies the consistency check: it reports where the resulting local classes agree, partially glue, disconnect, or become regime-sensitive. KET finally assembles the surviving local pieces into the user-facing artifact while preserving provenance. Thus, the paper’s main homotopy-localization layer does not require the reader to accept a large new theory stack at once. It requires only the workflow fact that Democritus organizes claims geometrically, checks their local compatibility, and aggregates the compatible pieces into an auditable causal database.

## 7. System Realization in Democritus and Cliff

Democritus realizes this perspective through a multi-stage document-analysis pipeline. A query first induces a retrieval cover, either from external corpora or a local document set. The system then extracts a root topic frontier, expands local discourse neighborhoods, generates candidate causal statements, constructs large numbers of local causal models, and compiles them into a causal SQL bundle (Csql) that supports provenance-preserving aggregation.

### Cliff as the Interface Layer

Cliff is the public-facing interface layer above this pipeline. The current public release, Cliff_CatAgi, describes it as a conscious interface or conscious layer sitting on top of a deeper Functor Flow engine. Operationally, that means three things. First, Cliff treats a user query as a routing problem: corpus-level Democritus analysis, workflow recovery, company comparison, and related synthesis tasks are exposed as distinct routes rather than collapsed into one monolithic prompt. Second, the heavier routes run as background workflows that gather evidence, normalize it into a shared structure, and synthesize bounded artifacts that can be reopened later. Third, optional external engines remain explicit dependencies rather than hidden assumptions: Democritus, BASKET/ROCKET, and related backends can live as sibling repositories or be located through environment variables.

This architectural separation matters for the present paper because the experimental objects are not just raw extracted triples. They are archived Cliff runs with route decisions, saved dashboards, and provenance-carrying Csql bundles. In that sense, Cliff is part of the experimental method. It provides the stable user-facing surface through which the localized claim classes, topic partitions, homotopy diagnostics, and gluing summaries become inspectable and reproducible. Table 7 summarizes the persisted output at each operational stage.

With that machinery in place, recent iterations of the system added four components that are central to the present paper.

1. **Homotopy localization.** The Csql bundle materializes canonical subject–relation–object classes and localizes paraphrastic claim families directly inside the database.
2. **Regime gluing.** A second family of views preserves canonical regimes and relation families, allowing the system to classify localized claims as multi-regime glued, regime-sensitive, or obstructed.
3. **Topic partitions.** Broad queries often retrieve heterogeneous document sets. Instead of forcing a flat synthesis, Cliff first decomposes the corpus into local topic covers.
4. **Archived interactive artifacts.** Runs are saved and indexed, so the full set of generated artifacts can be reopened later, reused in papers, and reproduced from the public code base.

These changes matter because they shifted the system from a pure extraction-and-summary pipeline to an experimental environment for studying causal language itself. Broad queries may show fragmented topic covers or many singleton partitions; focused queries may show strong multi-regime gluing; and borderline cases often appear as partially glued or disconnected simplicial families rather than falsely coherent backbones.

## 8. Experimental Framing

Our experiments are qualitative and systems-oriented rather than benchmark-driven. The aim is not to report a single scalar accuracy for causal extraction, but to study how a working extraction pipeline behaves under paraphrase variation, topic drift, and cross-document aggregation. We therefore focus on saved runs that are especially diagnostic of the central problem.

Three kinds of corpus behavior recur in practice.

1. **Focused corpora.** Narrow domains such as the Mediterranean diet tend to produce coherent local basins, interpretable topic partitions, and stable regime-glued claims.
2. **Broad but recoverable corpora.** Queries such as rising ocean temperatures can still produce useful gluing results once topic filtering and atlas sanitation are improved, even though the underlying domain remains multi-regime.
3. **Overbroad corpora.** Queries such as climate change or economic inflation often decompose into multiple local covers. In these cases, the atlas and partition layers are more informative than any single flattened global summary.

The core outputs we track are within-document and cross-document homotopy classes, the distribution of coherent, partially glued, and disconnected localized claim families, the counts of multi-regime glued, regime-sensitive, and obstructed claims, the topic-partition structure of the retrieved corpus, and the availability of supporting local causal graphs and manifold neighborhoods for the most important claims.

### 8.1. Evaluation Protocol and Ablations

The main object of evaluation remains the post-extraction layer: consolidation, localization, gluing, and artifact preservation. Its units of analysis are saved Democritus/Cliff runs and their archived Csql or public-bundle summaries. For each run, we record the document cover, the number of raw or near-raw causal mentions available in the archive, the number of localized or aggregated claim objects, the coherence or gluing labels produced by the system, and representative artifacts that allow a reader to audit the claims. To test the upstream extractor separately, we additionally report a frozen sentence/passage-level comparison against the public UniCausal baseline. This separates extraction quality from the later question of whether many extracted mentions can be normalized and glued coherently.

The qualitative case studies were validated by artifact audit rather than by an independent expert-annotation study. For each archived run used in the paper, we checked that the preserved source document identities, extracted claim records, localized claim classes, repeated-support summaries, and rendered artifacts were internally consistent and traceable through the saved Csql bundle or public example bundle. In the running example and the emperor-penguin PDF case, we also manually inspected the source article text and the strongest repeated causal backbones to confirm that the claims being discussed were supported by the article’s expressed causal discourse. This validation is sufficient for the paper’s diagnostic claim that Democritus organizes expressed causal discourse into auditable localized classes, but it is not a gold-standard accuracy evaluation of every extracted claim.

The resulting evaluation has two deliberately separate parts. First, the external comparison measures causal-passage classification and cause/effect span recovery before localization. Second, three lightweight ablations and a component-attribution table examine the later system. The first ablation is a *no-localization* contrast: count raw mentions or nearby variants before homotopy localization and compare them with the localized claims shown to the reader. The second is a *regime-projection* contrast: project away regime and polarity fields from the Csql views and ask which labels become unobservable. The third is a *flat-visualization* contrast: apply a generic manifold visualizer such as UMAP directly to extracted causal records and compare the result with the GT+DB workflow, which first builds meaningful local neighborhoods and then checks their compatibility. We also retain a transparent cue-pattern extractor, using markers such as *because*, *due to*, *caused by*, *leads to*, and *reduces*, as a low-capacity reference point [15,16].

Accordingly, the evidence should be read in tiers. The upstream extractor receives external benchmark evaluation; localization and regime preservation receive internal quantitative contrasts and artifact audit; and GT, DB, and KET receive component-attribution and diagnostic-visualization analysis rather than full replacement-module ablations. This tiered evaluation supports viability and inspectability of the categorical post-extraction workflow, but it does not yet establish externally validated correctness of every merge, non-merge, obstruction, or gluing decision.

### 8.2. External Causal-Relation Extraction Comparison

We froze the grouped AltLex test split distributed with UniCausal [17,18]. After grouping annotations by passage, the split contains 404 unique passages: 115 causal and 289 non-causal, with 127 annotated directed cause–effect pairs. Every method receives exactly the same untagged passage text. We report causal-passage precision, recall, and F1; token-level F1 separately for cause and effect spans; their macro-average; and exact and relaxed directed-pair F1. A relaxed pair counts as matched only when both the predicted cause and predicted effect overlap their corresponding gold spans. Because the public UniCausal tagger labels spans but does not pair multiple causes and effects, we pair its predicted spans in textual order solely for this secondary pair diagnostic; the classification and role-specific span scores do not depend on that convention.

The comparison includes three methods. The cue baseline predicts a causal passage when a member of a fixed causal-connective lexicon occurs. The UniCausal baseline uses its public sequence classifier and token classifier without fine-tuning on this split. The restricted Democritus adapter uses Kimi K2.5 [19] at temperature zero with reasoning disabled and a JSON-only contract. It may return any number of relations or an empty list, but every nonempty cause and effect must be copied verbatim from the passage; non-verbatim records are rejected before scoring. This benchmark bypasses document retrieval, topic expansion, localization, and gluing. It therefore evaluates the upstream extraction interface rather than the entire Democritus pipeline and avoids attributing post-extraction expansion to the base extractor. Table 8 reports the frozen comparison.

The comparison is competitive, but not a win for Democritus. UniCausal has the best classification F1 (59.2% versus 57.1%) and macro span F1 (43.6% versus 39.1%). Democritus finds more causal passages—80.9% recall versus 68.7%—but pays for that coverage with lower precision (44.1% versus 52.0%). In count terms, it produces 93 true positives, 118 false positives, and 22 false negatives, compared with 79, 73, and 36 for UniCausal. The restricted adapter completes all 404 requests without a request or JSON-parsing failure; six proposed relation records are rejected because at least one span is not an exact substring.

The exact-pair result exposes a more specific weakness. The Democritus adapter obtains no exact four-boundary pair matches, although 70 directed pairs match under the overlap criterion. Inspection shows that it commonly selects a semantically appropriate but broader clause than the compact annotated argument. Thus, the 35.8% relaxed-pair F1 should not be read as erasing the zero exact score: together they diagnose useful directional overlap coupled with poor annotation-boundary calibration. UniCausal, which is trained for this tag schema, retains a modest advantage even under the relaxed criterion. These results motivate span-boundary calibration and precision control as concrete upstream improvements while leaving the paper’s post-extraction claims to be tested by the localization and gluing experiments below. Table 9 begins those post-extraction tests with the no-localization contrast.

Table 10 then compares the archived single-document result with the cue-pattern baseline.

Table 11 isolates the effect of projecting away regime and polarity fields.

Table 12 gives the complementary structural-visualization contrast.

Table 13 attributes the observed effects to the implementation components.

Table 14 summarizes what was validated and what remains outside the evidence.

These ablations are deliberately small, but they sharpen the empirical interpretation of the case studies. Homotopy localization is valuable when many surface variants express the same normalized claim; it should have little effect when documents are genuinely study-specific, as in the red-wine collation. Regime preservation is valuable because it prevents a false sense of agreement: the system can distinguish a clean multi-regime claim from a sign conflict, a relation-family shift, or a cover that has drifted into neighboring subfields. The flat-visualization contrast adds a third point: GT+DB is not just syntactic decoration around ordinary embeddings, because the local neighborhoods and compatibility labels are what turn a dense projection into an auditable causal-discourse object. Table 13 further separates the contribution of the surrounding DB/GT/KET stack from the narrower localization and regime mechanisms studied most directly in this paper.

### 8.3. Mediterranean Diet as a Focused Corpus

The Mediterranean diet query is a good example of a focused corpus in which the localization machinery behaves well. In one ten-document run, the system reported no cross-document homotopy classes but a large number of within-document families, indicating that the studies shared mechanisms and regimes without repeating exactly the same backbone claims. The same run yielded 26 multi-regime glued claims, 4 regime-sensitive claims, and no obstructed claims. The most stable surfaces centered on the effect of Mediterranean diet adherence on gut microbiota composition and downstream microbial gene expression related to immune response and metabolism. The surviving archived Cliff checkpoint for this case preserves four selected source texts rather than a frozen final ten-document manifest, but those preserved texts still center on Mediterranean-diet microbiota and epigenetic mechanisms; Appendix C, Table A5, records that provenance explicitly, while Table 15 summarizes the run.

This case is instructive because it shows the desired behavior of the system. The topic cover remains tight, the regime-gluing layer surfaces a biologically meaningful local mechanism, and the remaining disconnected families are mostly clause-tail phenomena: one head claim branches into several downstream biological continuations. The absence of strict cross-document homotopy classes in this run should not be read as substantive disagreement. It more often means that neighboring papers describe compatible mechanisms at slightly different levels of granularity, so agreement persists locally and regime-wise without collapsing to a single repeated canonical sentence backbone across documents.

### 8.4. Red-Wine Study Collation as Topos-Style Gluing

To describe the gluing over causal claims from multiple documents using topos-theoretic constructions, we discuss two PubMed studies with overlapping but non-identical discourse about red wine and resveratrol: *Red Wine Consumption and Cardiovascular Health*, a cardiovascular-health article emphasizing endothelial function and cardioprotective pathways, and *Resveratrol: A Double-Edged Sword in Health Benefits*, a review emphasizing anti-inflammatory, endothelial, and mitochondrial mechanisms. These are the exact archived source papers paired in the saved study-collation run listed in Appendix C Table A5. Democritus first compiled each document into its own local causal atlas, yielding atlas *A* with 300 aggregated edges and atlas *B* with 268. The important point is that the two atlases do not share a flat ontology or identical sentence-level claims. They must be compared through a canonicalized interface of normalized subject–relation–object surfaces.

In this setting, the soft pullback plays the role of a match object over that interface. Instead of requiring literal equality between atlas edges, it searches for high-quality alignments after the normalization and localization steps described earlier. For this pair of studies, the pullback recovered two cross-study matches: a strong consensus around *resveratrol → mitochondrial biogenesis → enhanced energy metabolism*, and a weaker but still informative consensus around *resveratrol → endothelial function → nitric oxide/vascular relaxation*. These are precisely the kinds of cases where a purely lexical merge would either miss the overlap or flatten the distinction between a broad cardiovascular claim and a more specific mechanistic refinement.

**Figure 4:**
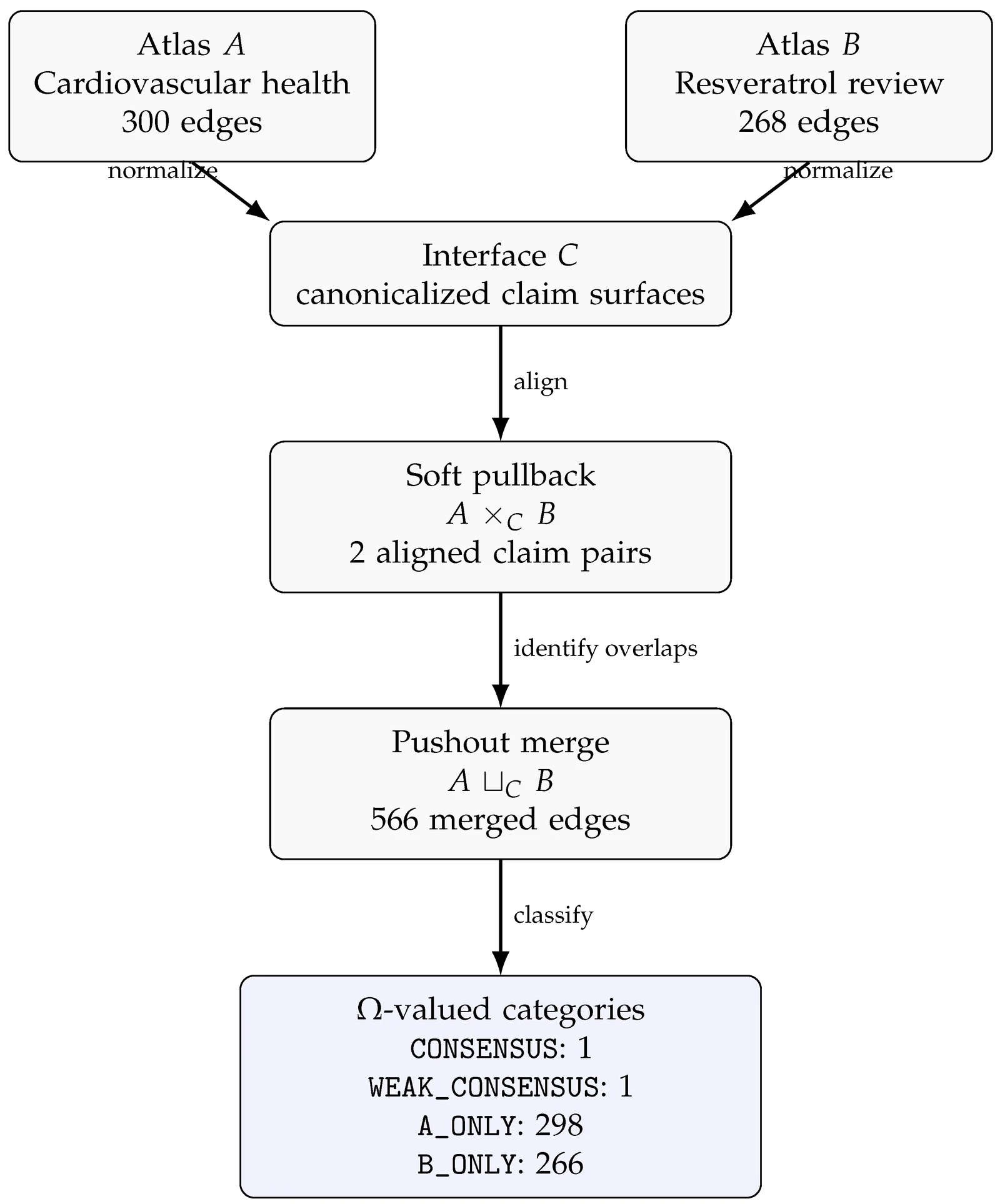
Topos-style study collation for the red-wine corpus. Local causal atlases are compared through a canonicalized interface, softly glued by pullback-style alignment, merged by pushout-style identification, and finally classified into logic-valued claim categories.

The complementary operation is pushout-style merging. Once the two overlapping claims are identified, the reconciled atlas is formed as the union of the two study atlases modulo those identifications. In the saved run, this yielded 566 merged edges, exactly 300+268−2, so the pushout preserves almost all study-specific structure while still creating a shared object for the two aligned mechanisms. This is important experimentally because it shows that gluing is not a lossy consensus summary. Most claims remain local to one study and are not forced into agreement just because the documents inhabit the same biomedical neighborhood. Figure 4 depicts the alignment and merge, and Table 16 reports the resulting counts.

This experiment also gives a concrete interpretation of the topos language. The pushout atlas carries a simple truth-value assignment χ:Claims→Ω, where Ω is the four-valued space {CONSENSUS, WEAK_CONSENSUS, A_ONLY, B_ONLY}. In computational terms, χ is a provenance-aware classifier on merged claims. In categorical terms, it is a small subobject classifier that lets us isolate the consensus subobject while preserving unmatched claims as local sections that failed to glue. That is exactly the kind of reasoning we want from Democritus: identify what genuinely descends across documents, retain what stays study-specific, and expose both outcomes in auditable form.

### 8.5. A Single-Document PDF Experiment: Emperor Penguins

A complementary focused experiment comes from a single Washington Post PDF on emperor penguins, Antarctic sea ice, and warming ocean currents, archived from Sarah Kaplan’s 9 April 2026 article “Emperor penguins have just been declared endangered” (https://www.washingtonpost.com/climate-environment/2026/04/09/emperor-penguin-endangered-antarctic-fur-seal/ (accessed on 10 April 2026)). Because the retrieved corpus contains only one document, there is no cross-document agreement to measure; the diagnostic question becomes whether one article still supports a stable internal causal neighborhood rather than a flat summary. In the preserved Csql archive, Democritus stores 329 claim records over 18 domains, 316 aggregated edges, and 264 homotopy-localized claim classes. Of these localized classes, 20 receive repeated within-document support, and the two strongest repeated classes each consolidate 16 raw claim records. The dominant repeated backbone linked rising ocean temperatures to krill moving to deeper waters and thereby reducing prey availability for emperor penguins and Antarctic fur seals; a second recurring chain linked global warming to Antarctic sea-ice loss, breeding-habitat degradation, and increased chick mortality. The archive retains the single acquired PDF identity for this case study, and Appendix C, Table A5, records that provenance. Table 17 summarizes the single-document run.

This kind of run is useful because it separates two different notions of stability. Cross-document homotopy cannot appear when there is only one source, but within-document paraphrase collapse still matters: the system can show whether an article repeatedly returns to the same mechanism under slightly different formulations or instead fragments into unrelated local claims. In this case, the repeated support surfaces are ecologically coherent, which makes the run a clean demonstration that the homotopy-localization layer is already informative before any multi-document gluing is attempted.

### 8.6. Rising Ocean Temperatures as a Multi-Regime Corpus

An archived run launched from the query for five recent studies on rising ocean temperatures shows a complementary failure mode: the corpus is scientifically recognizable as a single domain, but at the homotopy-localization layer it still behaves mostly as a collection of local causal patches rather than as a document set with repeated cross-document backbones. In the preserved archive, the retrieval cover ultimately contains six acquired documents, and the run produced 0 cross-document classes and 1570 within-document families, of which 1522 were coherent, 7 partially glued, and 41 disconnected. In other words, most of the stability in this corpus lives inside individual articles rather than across them. That is already a useful diagnosis. It tells us that the retrieval cover is semantically related, but the extracted mechanisms are often expressed at different granularities, under different regimes, or in different scientific subdomains. Appendix C Table A5 makes the cover drift visible: alongside ocean-warming studies, the saved run preserved neighboring sources on cyclone intensification, heat-related mortality, mantle melting, and phytoplankton adaptation.

The disconnected and partially glued families make this visible in an unusually concrete way. One of the strongest local patches concerns the North Atlantic mechanism *weakening of the subpolar gyre → increased inflow of warm saline subtropical waters into the North Atlantic*. Although this family is confined to a single study, it has 25 vertices, 164 edges, 821 verified triangles, and a multiway closure ratio of 0.87, so its observed compatibility complex is extremely dense. The archived implementation calls this statistic a “horn-fill ratio,” but mathematically it is a finite candidate-triangle closure statistic on $K_{\left[c\right]}^{\mathrm{obs}}$, not a horn-filling test in the nerve of a groupoid. By contrast, the freshwater-stratification family *freshwater influx from rivers and rainfall → increased ocean stratification* appears only partially glued, with 12 vertices, 19 edges, and a closure ratio of 0.258, indicating wording drift and context-sensitive elaboration. Other disconnected families wander further afield into heat-mortality adaptation discourse and mixotrophic-phytoplankton ecology, showing that even a six-document archived cover can mix climate, oceanography, and ecological-mechanism subcovers. Table 18 summarizes the homotopy-localization results for this run.

This is the kind of output we regard as especially valuable. A standard summarizer would compress the corpus into a narrative about warming oceans. The homotopy view instead shows that the corpus contains several locally stable causal neighborhoods without yielding many exact cross-document identifications. That distinction matters scientifically: some mechanisms are robust but article-specific, some are only partially glued because the clause tails drift across regimes, and some apparent neighbors are really signals that the retrieved cover has broadened into adjacent subfields.

### 8.7. Broad Corpora and Topic Partitions

**Figure 5:**
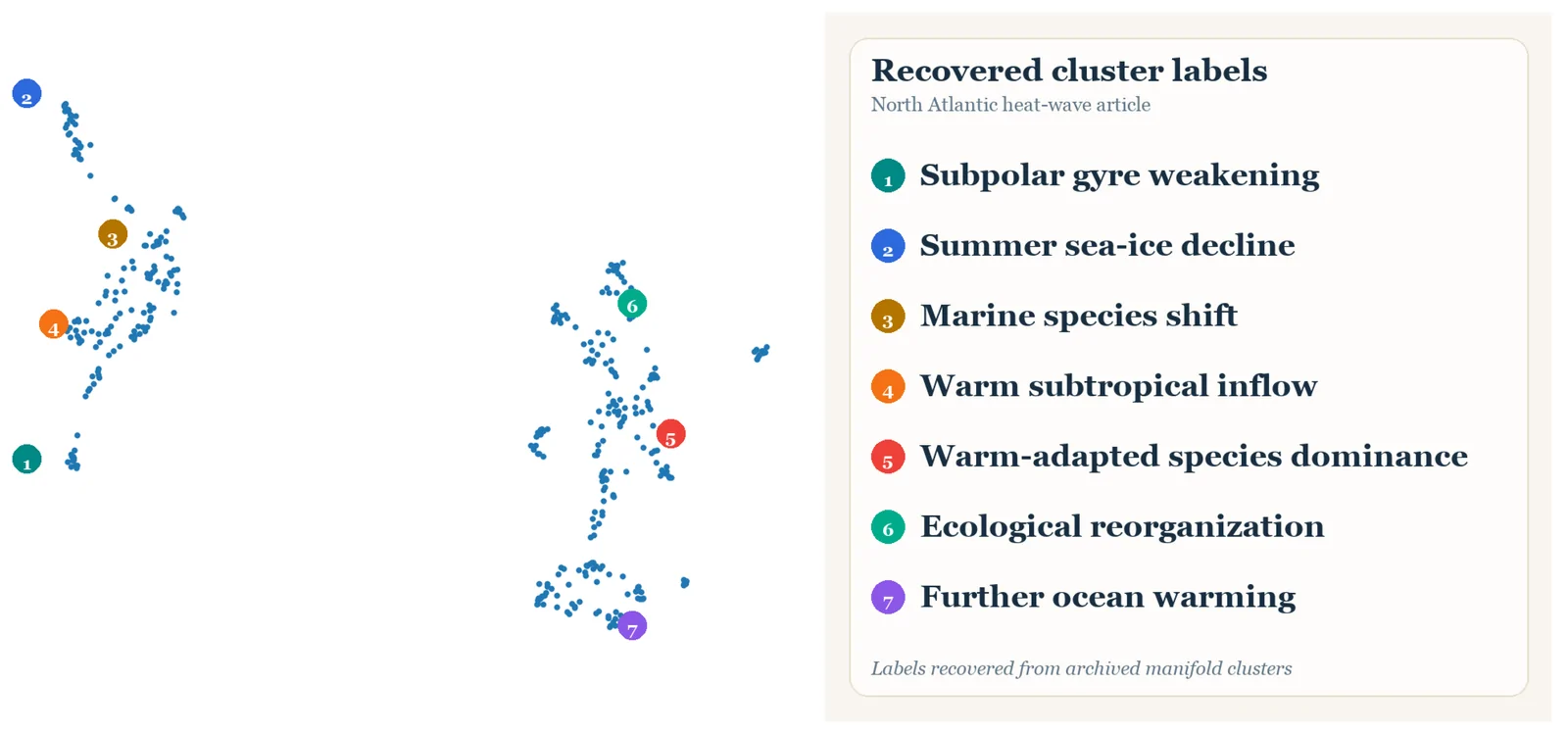
Archived two-dimensional relational manifold for the North Atlantic heat-wave article from the same ocean-temperature run, with numbered cluster labels recovered from the saved manifold metadata. The largest neighborhoods correspond to subpolar-gyre weakening, summer sea-ice decline, warm-adapted species shifts, and ecological reorganization, making the dominant local mechanism basins visible even before any cross-document gluing succeeds.

The broad-climate experiments revealed another lesson. When the base query is too broad, the homotopy and regime layers should not be read before the cover itself is understood. Early runs often wandered into narrow basins such as malaria, while later retrieval and topic-atlas improvements yielded broader covers across climate-health, water systems, adaptation policy, coral bleaching, and ocean warming. Even so, many broad corpora remained collections of local patches rather than a single cleanly glued object. This is why topic partitioning became a first-class diagnostic. Figure 5 shows the local mechanism basins for one archived ocean-temperature article.

Singleton topic partitions are especially informative in this setting. They do not automatically signal extraction failure; often they mark documents that enter the retrieved cover through a shared high-level query but then instantiate a mechanism, regime, or discourse frame that no other document in the run matches closely enough to glue. Read together with the absence of strict cross-document homotopy classes in otherwise coherent corpora, these singleton partitions show why cover structure must be interpreted before global agreement claims are made: local causal neighborhoods can be internally stable and scientifically relevant even when the corpus as a whole never forms one perfectly repeated cross-document backbone.

## 9. Related Work

This paper sits at the intersection of several literatures: classical causal inference, causal relation extraction from text, causal knowledge-graph construction, and recent attempts to use large language models for causal reasoning. Our position relative to each is slightly different, and it is useful to state that explicitly.

Relative and homotopical categories study mathematical objects up to a declared class of weak equivalences rather than only up to literal isomorphism [12]. The present use of that language is deliberately small: normalization induces the weak equivalences, localization gives their universal inversion, and the observed compatibility complex records which finite coherence checks have actually been performed. We do not impose a model structure on raw language, nor do we claim that the implementation computes an (∞,1)-categorical localization.

The closest categorical predecessor is the higher algebraic K-theory of causality [11], which studies classifying spaces built from observationally equivalent formal causal models represented through cPROP functors. The present paper acts one level earlier. Its relative category contains causal *discourse* objects, and Proposition 3 states the compatibility needed for a grounded discourse functor to descend into a localization of formal causal models. Thus the earlier work studies the topology within causal-model equivalence classes, whereas Democritus studies how language-derived evidence can be stabilized before entering those classes.

Relative to classical causal inference, our work is complementary rather than competitive. Modern causal inference studies identifiability, adjustment, intervention, equivalence classes of causal models, and structure learning from observational or experimental data [3,4,5,6,7,8,9]. We do not attempt to replace those methods with language alone. Instead, we study the prior layer at which causal discourse is produced, compared, and consumed. In this sense, Democritus is closer to a causal hypothesis compiler or diagnostic front end than to a structure-learning algorithm over data.

Relative to causal relation extraction and scientific information extraction, our setting is broader and more artifact-centered. Existing work on causal relation extraction, scientific IE, and claim verification usually targets sentence-level or passage-level extraction accuracy [15,16,20,21]. The benchmark and corpus lineage includes nominal cause–effect relation classification, alternative lexicalizations of causality, event-causality corpora, biomedical causality resources, financial causality shared tasks, and scientific information-extraction benchmarks [17,22,23,24,25,26,27]. Those systems and resources are valuable, but they do not usually address what happens after extraction when thousands of overlapping causal mentions must be consolidated, compared across documents, and audited for disagreement or regime dependence. The homotopy-localization layer in Democritus is aimed precisely at that post-extraction instability.

Section 8.2 supplies a controlled comparison for the upstream layer using the native UniCausal/AltLex annotation schema. It should not be read as a state-of-the-art claim: the public UniCausal baseline remains stronger overall, the comparison covers one English split, and its span annotations do not evaluate cross-document localization or merging. A complementary benchmark is still needed for the post-extraction layer—in particular, clusters of paraphrastic and regime-sensitive claims with independently annotated merge, non-merge, and gluing decisions.

Relative to corpus-scale causal claim resources, the closest comparison is the Testing Causal Claims (TCC) effort, which aggregates causal claims from a large economics corpus [28]. TCC emphasizes breadth, method metadata, and large-scale indexing of causal statements across a discipline. Democritus emphasizes something different: local causal modeling within documents, explicit paraphrase collapse, regime-gluing diagnostics, and interactive artifact preservation. The two approaches are complementary. A resource like TCC offers a broad compiled layer of causal claims, whereas Democritus offers a way to study how such claims cohere, fragment, or fail to glue inside narrower local covers. We also built an older specialized Cliff variant with a dedicated tcc_atlas route that queries the TCC atlas/Csql store directly rather than retrieving documents on the fly; although that branch predates the current public Cliff, it demonstrates that the same interface layer can scale to much larger curated claim repositories.

Relative to knowledge graphs and GraphRAG, our system again targets a different object. Knowledge graphs and graph-augmented retrieval systems are effective at representing and retrieving asserted relationships [29,30]. But they are not usually designed to distinguish paraphrastic duplication from genuine cross-regime conflict, or to preserve a notion of support grounded in many local causal models. In Democritus, the graph is not just a storage substrate. It is part of an experimental object that includes equivalence classes, support provenance, and explicit gluing diagnostics.

Finally, there is a fast-growing literature on whether large language models can perform causal reasoning or assist in causal discovery. Some of that work probes causal competence directly; some uses language models to propose structures or explanations. Our stance is intentionally more conservative. We use language models as discourse compilers and local hypothesis generators, not as authoritative causal reasoners. The emphasis is on what can be stabilized *after* generation through normalization, localization, scoring, and aggregation. In that sense, the current paper is less a claim about the innate causal intelligence of LLMs than a claim about how to build a better causal-language analysis environment around them.

## 10. Discussion and Limitations

Several lessons emerge from these experiments. First, paraphrase handling cannot be treated as a cosmetic post-processing step. It changes which claims accumulate support, which mechanisms appear recurrent, and whether a corpus seems coherent at all. Second, gluing failures are often scientifically useful. A regime-sensitive or obstructed claim is not simply an error; it may indicate that one mechanism changes sign across contexts, that different studies use relation families at different levels of strength, or that the apparent agreement is only local. Third, retrieval and synthesis must be studied together. Many apparent failures of causal extraction begin one layer earlier, as failures of the retrieved cover. The red-wine collation experiment adds a fourth lesson: the topos vocabulary is not merely decorative. Pullback-style alignment, pushout-style merging, and logic-valued claim categories correspond to concrete database operations that preserve provenance while separating true cross-study consensus from study-specific hypotheses.

The present work also has clear limitations. We do not claim that the extracted claims are ground-truth causal facts, only that they are structured and diagnostically useful summaries of causal discourse. The current homotopy layer is coarse: many disconnected families are really head-tail clause-segmentation problems, and $K_{\left[c\right]}^{\mathrm{obs}}$ is a finite record of tested compatibility rather than a derived invariant. The paper establishes a homotopical *base*-repair problem, not infinitesimal non-compositionality; no tangent functor on causal discourse is defined. Proposition 3 is also conditional: the live system does not automatically construct the grounded relative functor Φ, and normalized claims remain hypotheses until variables, mechanisms, and causal assumptions are supplied. Finally, nothing here proves that the discourse localization induces a map of algebraic K-theory spaces.

The experiments combine one extraction benchmark with systems-oriented case studies. The 404-passage UniCausal/AltLex comparison evaluates the restricted upstream extractor, but it remains small, English-only, and tied to one annotation schema. It also shows a real boundary-calibration problem and a precision–recall tradeoff rather than benchmark superiority. The strongest empirically supported claims are therefore that the upstream extraction interface is externally comparable, that the saved artifacts are internally consistent and provenance-preserving, and that the reported ablations expose concrete losses when localization or regime information is removed. As Table 14 states, the later localization and gluing case studies were validated through artifact audit, provenance checks, and manual inspection of representative high-support claims, not through a formal expert annotation campaign or user study. In particular, no independent annotators judged the merge and non-merge decisions. Although Table 13 separates the observed roles of homotopy localization, regime gluing, DB, GT, and KET, only the localization and regime layers are ablated quantitatively in the strict sense. The DB and GT rows are supported by diagnostic removal and flat-visualization contrasts over saved artifacts, while KET is treated as an artifact-level contrast; they are not yet independent no-DB, no-GT, and no-KET reruns with controlled replacement modules. A fuller engineering evaluation should add diverse causal-extraction corpora, expert-labeled merge decisions, and controlled replacements for the DB, GT, and KET components.

### Future Evaluation

The most important next step is an independent evaluation of the post-extraction layer itself. Such a benchmark should contain paraphrastic, partially overlapping, and regime-sensitive causal claims annotated by independent experts for merge, non-merge, and gluing decisions. It should evaluate whether localization reduces paraphrase inflation without over-merging distinct mechanisms, whether regime labels prevent false consensus, and whether pullback/pushout-style collation preserves provenance while exposing genuine cross-study overlap. A second direction is temporal evaluation: once document-local causal snapshots can be compared reliably, yearly or regime-indexed corpora could be tested for coherent trajectories and drift. Those extensions remain future work; the present paper evaluates the static causal-discourse setting and treats the categorical machinery as a specification for auditable post-extraction diagnostics.

## 11. Conclusions

The main challenge in causal extraction from language is not merely finding candidate cause–effect records. It is deciding when different textual realizations should be identified, when they should remain distinct, and how local agreements should compose across documents and regimes. We formulated that task as homotopical repair of a non-compositional causal-discourse sketch. Normalization induces a relative category and factors through its localization; localized sketch factorization separates strict agreement from agreement up to semantics-preserving variation; and a relative grounding functor, when supplied, maps this discourse-level construction into observational-equivalence classes of formal causal models. In the implemented system, homotopy localization reduces paraphrase inflation, observed compatibility records finite coherence decisions, regime gluing distinguishes clean agreement from obstruction, pullback- and pushout-style views make reconciliation auditable, and archived artifacts preserve the resulting evidence. The result is not a causal-discovery oracle or a computation of higher K-theory, but a mathematically specified and operationally inspectable bridge from unstable causal language toward formal causal-model spaces.

## Figures and Tables

**Table 1:** A simplified extraction trace from the emperor-penguin case. The raw mentions are not yet evidence units; they must first be normalized and compared.

ID	Raw Causal Mention	Immediate Extraction
$m_{1}$	Rising ocean temperatures lead krill to move into deeper waters.	rising ocean temperatures → krill move deeper
$m_{2}$	Warming waters drive krill away from surface feeding zones.	warming waters → krill leave surface zones
$m_{3}$	Reduced near-surface krill lowers food availability for emperor penguins.	less surface krill → reduced penguin food
$m_{4}$	Loss of Antarctic sea ice degrades emperor-penguin breeding habitat.	sea-ice loss → breeding-habitat degradation
$m_{5}$	Breeding-habitat degradation increases chick mortality.	habitat degradation → increased chick mortality

**Table 2:** Normalized causal fields. In the implementation, candidate fields are produced by the extraction workflow and then canonicalized into controlled relation, polarity, temporal, regime, qualifier, and provenance fields stored in the Csql bundle.

ID	Cause	Effect	Relation	Sign	Time	Regime
$m_{1}$	ocean temperature increase	krill depth shift	displacement	positive	ongoing warming	Antarctic marine ecology
$m_{2}$	ocean temperature increase	krill surface availability	reduction	negative	ongoing warming	Antarctic marine ecology
$m_{3}$	surface krill availability	emperor-penguin food availability	support	positive	foraging season	Antarctic food web
$m_{4}$	Antarctic sea-ice loss	breeding-habitat quality	degradation	negative	seasonal to long-term	emperor-penguin breeding
$m_{5}$	breeding-habitat degradation	chick mortality	increase	positive	breeding season	emperor-penguin breeding

**Table 3:** Weak-equivalence decisions in the running example. The system merges paraphrases only when normalized causal content is preserved; related mechanism steps are linked without being identified.

Comparison	Decision	Output Class	Reason
$m_{1}$ vs. paraphrase of $m_{1}$	Merge	localized class $\left[c_{1}\right]$	Same canonical cause/effect, displacement relation, positive polarity, warming scope, and Antarctic marine regime.
$m_{1}$ vs. $m_{2}$	Link but do not merge	mechanism chain $\left[c_{1}\right]\to \left[c_{2}\right]$	Closely related ecological mechanism, but different normalized effect and relation family.
$m_{4}$ vs. $m_{5}$	Do not merge	chain $\left[c_{4}\right]\to \left[c_{5}\right]$	The effect of one claim becomes the cause of the next; merging would collapse a causal pathway.
Sea-ice loss improves habitat vs. $m_{4}$	Conflict/obstructed	regime-sensitive or obstructed family	Same surface entities but opposite polarity, requiring separation unless a distinct regime explains the sign change.

**Table 4:** Normalization fields used to approximate weak equivalence in Democritus. The mathematical notation treats normalization as a functor; the implementation realizes it as a schema, canonicalization procedure, and set of stored comparison decisions in Csql.

Field	Implementation Role	Effect on Equivalence
Cause *X*	Canonical cause phrase or entity-like expression.	Must match after canonicalization, synonym normalization, and allowed granularity adjustment.
Effect *Y*	Canonical effect phrase or entity-like expression.	Must match after canonicalization; mechanism chains are linked rather than merged when one claim’s effect becomes another claim’s cause.
Relation family *r*	Controlled relation type such as increase, decrease, enable, inhibit, displace, mediate, or associate.	Must be compatible; broad relation words such as “affects” are not merged with directional claims unless polarity and mechanism are resolved.
Polarity *s*	Positive, negative, neutral/associative, or unknown sign.	Opposite signs block equivalence unless separated by an explicit regime label.
Temporal scope τ	Time horizon, season, experimental window, historical period, or unknown.	Incompatible temporal scopes prevent merging; nested scopes may be linked by refinement.
Regime κ	Domain, population, geography, experimental condition, or contextual frame.	Claims are merged only when regimes are compatible; otherwise they are reported as regime-sensitive or obstructed.
Qualifiers *q*	Hedging, confidence, provenance, evidence source, and extraction notes.	Contradictory qualifiers block automatic merging; provenance is preserved even after merging.

**Table 5:** Practical role of the categorical layer. The implementation approximates these objects with normalized keys, stored comparison decisions, gluing labels, and Csql provenance tables; the empirical case studies audit those approximations rather than proving that the formal objects have been fully computed.

Categorical Object	Operational Role in Democritus	What Is Lost Without It
Weak equivalences	Declare which mention rewrites preserve normalized causal content.	Similarity clustering has no principled reason to block merges across polarity, temporal scope, or regime.
Localization	Aggregate evidence after admissible paraphrase variation is inverted.	Support counts remain tied to unstable wording, producing paraphrase inflation or arbitrary duplicate removal.
Sketch repair	Test whether document-local claims compose after normalization-preserving variation.	A corpus can be summarized as a bag of clusters, but failures of coherent assembly are not distinguished from ordinary sparsity.
Pullback-style alignment	Compare two atlases through a canonical interface of normalized claim surfaces.	Cross-study matches become ad hoc pairwise joins rather than auditable overlap witnesses.
Pushout-style merge	Form a provenance-preserving union modulo accepted overlap claims.	Merged atlases risk either double-counting shared mechanisms or erasing study-specific claims.
Observed compatibility complex	Record finite pairwise and multiway compatibility decisions inside a localized family.	Coherent, partially glued, and disconnected families collapse to a single similarity score.

**Table 6:** Operational reading of DB, GT, and KET in the current Democritus workflow.

Role	Input	Processing Step	Output Used Here
Geometric organization (GT)	Extracted causal statements, relation labels, topics, and document-local models	Build neighborhoods in which nearby claims reflect mechanistic and regime similarity rather than only word overlap	Topic partitions, manifold views, nearest-neighbor expansions, and local comparison neighborhoods
Consistency checking (DB)	Local states, candidate overlaps, localized classes, and regime projections	Compare whether different local views agree when projected through shared causes, effects, relations, or regimes	Coherent, partially glued, disconnected, regime-sensitive, or obstructed labels
Aggregation (KET)	Document-local artifacts, localized classes, gluing labels, scores, and provenance links	Assemble many partial outputs into a bounded corpus-level synthesis without erasing source support	Dashboards, corpus summaries, candidate backbone claims, and causal database views

**Table 7:** Operational pipeline used in the current Democritus/Cliff implementation. The table separates extraction, normalization, localization, regime gluing, and artifact generation because these are distinct failure points in the experiments.

Stage	Input	Operation	Persisted Output
Query planning	User query or local file path	Normalize request, choose retrieval route, set target document count	Query plan JSON
Retrieval/manifest	Search backend, file path, URL, or manifest	Acquire or select documents and record source metadata	Corpus manifest and selected-document metadata
Document intake	PDFs, HTML, or Markdown summaries	Extract text and create document-local run records	Per-document run directory
Topic discovery	Document text	Recover root topics and local discourse neighborhoods	Topic frontier, topic graph artifacts
Mention extraction	Text neighborhoods	Generate causal statements, questions, and triples	JSONL claim/triple records
Normalization	Candidate mentions	Canonicalize subject, relation, polarity, object, domain/regime, and surface form	Csql claims table
Homotopy localization	Normalized claims	Group mentions by canonical causal content and surface variants	Homotopy-localized claim view
Regime gluing	Localized claims with domains and polarities	Classify surfaces as single-regime, multi-regime glued, regime-sensitive, or obstructed	Regime-gluing surface view
LCM/manifold artifacts	Extracted triples and topics	Build local causal models, manifolds, and visual summaries	LCM galleries, manifold plots, dashboards

**Table 8:** Frozen causal-relation extraction comparison on 404 grouped AltLex/UniCausal passages. Values are percentages. This table evaluates the upstream extraction interface only; localization and gluing are evaluated separately below. Bold values indicate the best result in each metric column.

Method	Cls. P	Cls. R	Cls. F1	Cause F1	Effect F1	Span Macro F1	Exact Pair F1	Relaxed Pair F1
Cue-pattern baseline	50.0	22.6	31.1	—	—	—	—	—
UniCausal public baseline	52.0	68.7	**59.2**	**40.8**	**46.5**	**43.6**	**20.5**	**37.3**
Democritus, restricted Kimi K2.5	44.1	**80.9**	57.1	37.1	41.1	39.1	0.0	35.8

**Table 9:** Minimal no-localization contrasts. Localization reduces paraphrase inflation when repeated variants are present; the red-wine collation intentionally preserves mostly study-specific structure.

Run/Bundle	Before Localization	After Localization	Interpretation
Emperor penguin single PDF	329 preserved claim records	264 localized classes	19.8% reduction; full Csql archive.
Ocean-warming public subset	249 claims plus variants	99 localized claims	60.2% reduction; compact public bundle.
Red-wine study collation	568 atlas edges	566 pushout edges	0.4% reduction; most claims remain study-specific.

**Table 10:** Single-document comparison with a pattern-based causal-relation extraction baseline on the archived Washington Post emperor-penguin PDF. The baseline detects explicit causal cues; the Democritus rows report post-extraction expansion, normalization, and localization.

Method on WaPo Penguin PDF	Count	Interpretation
Cue-pattern baseline, raw PDF text	8 cue-bearing sentences	Includes advertisement or page-boilerplate false positives from the archived PDF text.
Cue-pattern baseline, article body after removing obvious boilerplate	5 cue-bearing sentences	Recovers the explicit global-warming, sea-ice, chick-mortality, krill-depth, and ocean-circulation relations.
Democritus raw preserved relation records	329 claim records	Expands the article into topic-conditioned causal statements and local causal neighborhoods.
Democritus homotopy-localized classes	264 localized classes	Collapses repeated paraphrases while preserving relation, polarity, domain, and provenance fields.
Democritus localized classes with repeated support	20 classes	Surfaces repeated backbones; the strongest two classes each consolidate 16 raw claim records.

**Table 11:** Projection ablation for regime and polarity fields. The preserved schema can distinguish multi-regime corroboration, regime sensitivity, and obstruction; a collapsed schema can still aggregate text, but it cannot say why a family glued or failed to glue.

Run	Regime/Polarity Preserved	After Regime Projection
Mediterranean diet	26 multi-regime glued claims, 4 regime-sensitive claims, 0 obstructed claims	The same 30 non-single-regime surfaces become undifferentiated merged surfaces; regime sensitivity is no longer reportable.
Ocean temperatures	1522 coherent, 7 partially glued, and 41 disconnected within-document families over a drift-prone cover	Cover drift remains visible only as topical fragmentation; regime-conditioned reasons for partial gluing are hidden.
Running penguin example	Opposite-polarity sea-ice/habitat claims are blocked or marked as obstructed unless separated by regime	Surface entities alone would allow an erroneous merge of habitat-improvement and habitat-degradation claims.

**Table 12:** Structural visualization ablation. A generic projection method such as UMAP is useful for exploratory visualization, but applied directly to raw causal records it does not supply the localized neighborhoods and gluing diagnostics needed by the present workflow.

Visualization Condition	Structure Available to the Viewer	Operational Consequence
Raw extracted records with a generic UMAP projection	A single low-dimensional proximity plot over unlocalized cause–effect records	Produces a dense “hairball” view in which repeated paraphrases, regime shifts, and local incompatibilities are visually entangled.
Localized records without DB labels	Canonicalized claim classes and fewer duplicate surface forms	Reduces paraphrase clutter, but does not explain which neighborhoods cohere, partially glue, or remain disconnected.
GT+DB workflow used here	Topic/manifold neighborhoods plus coherence, partial-gluing, disconnected, and regime-sensitive labels	Produces inspectable local patches and auditable failure modes; the visualization is tied to the same compatibility tests used in the causal database artifacts.

**Table 13:** Component-level attribution for the current implementation. The first two rows are direct quantitative ablations. The DB and GT rows include diagnostic or structural visualization contrasts, while the KET row is an artifact-level contrast. These rows should not be read as full retraining-based module ablations.

Component	Contrast Used Here	Observed Contribution	Ablation Status
Homotopy localization	Raw or near-raw mentions vs. localized claim classes	Penguin: 329→264 classes. Ocean public subset: 249→99 displayed localized claims.	Direct quantitative ablation; Table 9.
Regime gluing	Project away regime and polarity fields	Mediterranean: 26 multi-regime glued plus 4 regime-sensitive surfaces collapse to 30 undifferentiated surfaces.	Direct projection ablation; Table 11.
Diagrammatic Backpropagation (DB)	Remove consistency/gluing labels after localization	Coherent, partially glued, and disconnected labels become unavailable; ocean becomes an untyped set of within-document families.	Diagnostic label projection plus Table 12.
Geometric Transformers (GT)	Replace topic/manifold neighborhoods with a flat cover or generic projection	Manifold neighborhoods, nearest-neighbor expansions, and topic partitions disappear.	Structural visualization contrast; full no-GT reruns are future work.
Kan Extension Transformers (KET)	Compare document-local outputs with corpus-level synthesis artifacts	Local claims remain, but dashboards, corpus summaries, Csql views, and provenance-preserving aggregates are not assembled.	Artifact-level contrast; not an independently benchmarked interchangeable module.

**Table 14:** Validation status of the extraction benchmark and qualitative case studies. The paper externally evaluates the restricted upstream extractor and validates artifact consistency and source traceability, but does not claim expert-validated merging or ground-truth causal correctness.

Validation Question	What Was Checked	What Remains Unvalidated
Source provenance	Archived document identities, selected-document manifests, and public bundle summaries were checked for the runs reported in the paper.	Some older runs preserve incomplete publisher metadata; the Mediterranean archive is a checkpoint rather than a frozen final manifest.
Upstream causal extraction	Frozen evaluation on 404 grouped AltLex/UniCausal passages against the public UniCausal models and a cue baseline; Table 8.	One English benchmark split does not establish robustness across domains, languages, document lengths, or model families.
Claim support	Representative high-support claims were traced back to saved claim records and, for the WaPo penguin case, to the article text.	No exhaustive manual annotation of all extracted claims was performed.
Merge decisions	Localization was audited through normalized fields, support counts, and representative equivalence decisions.	No independent expert panel labeled whether every merge or non-merge was correct.
Gluing diagnostics	Regime and polarity labels were checked through saved Csql views and projection ablations.	The gluing labels are system diagnostics, not externally validated causal facts.
User-facing usefulness	Artifacts were inspected for auditability, provenance, and interpretability.	No user study or expert usability study was conducted.

**Table 15:** Homotopy and regime-gluing summary for a focused ten-document Mediterranean diet run. The corpus shows strong local coherence and essentially no polarity-level obstruction.

Mediterranean Diet Run	Count
Cross-document classes	0
Within-document families	978
Coherent classes	958
Partially glued classes	2
Disconnected classes	18
Multi-regime glued claims	26
Regime-sensitive claims	4
Obstructed claims	0

**Table 16:** Summary of the red-wine study-collation experiment. The four-valued classifier Ω separates the merged atlas into consensus and study-specific regions rather than collapsing everything into a single undifferentiated backbone.

Object/Category	Count	Interpretation
Edges in atlas *A*	300	Cardiovascular red-wine discourse
Edges in atlas *B*	268	Resveratrol-review discourse
Soft pullback matches	2	Cross-study aligned mechanisms
Pushout edges	566	Union modulo matched overlaps
CONSENSUS	1	High-similarity glued claim
WEAK_CONSENSUS	1	Lower-similarity but auditable glued claim
A_ONLY	298	Study-specific claim retained from atlas *A*
B_ONLY	266	Study-specific claim retained from atlas *B*

**Table 17:** Single-document summary for the archived Washington Post emperor-penguin PDF experiment. Even without cross-document evidence, the run exhibits repeated internal support for a small number of central causal chains.

Emperor Penguin PDF Run	Count
Documents	1
Extracted claim records	329
Domains	18
Aggregated edges	316
Homotopy-localized classes	264
Localized classes with repeated support	20
Maximum support for one localized class	16

**Table 18:** Homotopy-localization summary for the archived rising-ocean-temperatures run. The original query asked for five studies, but the preserved retrieved cover contains six acquired documents. The absence of cross-document classes, together with the large number of coherent within-document families shows that the corpus forms a related cover without collapsing to a single repeated global backbone.

Rising Ocean Temperatures Run	Count
Documents	6
Cross-document classes	0
Within-document families	1570
Coherent classes	1522
Partially glued classes	7
Disconnected classes	41

## Data Availability

The original contributions presented in this study are included in the article. Further inquiries can be directed to the corresponding author.

## Appendix A. System and Database Appendix

This paper is centered on causal extraction from language rather than on the full Csql database theory, but the database layer remains important because it is where localized claim classes, regime summaries, and provenance-preserving aggregation are materialized. In the current implementation, each document run produces a collection of local causal models, their scores, rendered graph views, and a Csql bundle that stores canonical claims, localized claim families, and support tables linking aggregate claims back to individual local models and source documents.

**Table A1:** Implementation map for the revised paper. File names refer to the public CLIFF_CatAgi/functorflow_v3/ package unless otherwise noted.

Implementation Object	Representative File or Table	Role in the Experiments
Route and orchestration layer	functorflow_v3/cliff.py, cliff_worker.py	Parses the natural-language query, selects the Democritus route, records run metadata, and writes the route-specific output directory.
Query-driven corpus acquisition	democritus_query_agentic.py	Builds or loads the document cover, writes selected-document manifests, and launches per-document analysis.
Per-document causal extraction	democritus_agentic.py, democritus_batch_agentic.py	Produces root topics, causal questions, causal statements, relational triples, local causal models, and manifold artifacts.
Frozen extraction benchmark	Public repository path: experiments/causal_extraction_benchmark/benchmark.py	Runs the cue, public UniCausal, and restricted Democritus extractors and computes classification, role-span, and directed-pair metrics.
Normalization and localization	causal_homotopy.py, csql_bundle.py	Canonicalizes surface claims and materializes homotopy-localized and regime-localized views.
Csql storage	claims, homotopy_localized_claims, regime_gluing_surfaces	Stores raw claim records, localized classes, support counts, regime summaries, and provenance links.
Corpus synthesis and dashboards	democritus_corpus_synthesis.py	Reads Csql views and saved artifacts to render corpus summaries, topic partitions, and gluing diagnostics.

**Table A2:** Computational complexity of the implemented pipeline. The dominant practical cost in live runs is upstream LLM and retrieval latency; the localization and Csql stages are comparatively lightweight for the document counts studied here.

Stage	Asymptotic Driver	Comment
Document retrieval and intake	O(D) network and parsing calls	*D* is the number of selected documents; wall time is dominated by network, PDF parsing, and remote API latency.
Topic and statement generation	O(DTQS) LLM calls in the worst case	*T* is topics per document, *Q* causal questions per topic, and *S* statements per question; in practice these are capped by depth-limit, max-total-topics, and statements-per-question.
Claim normalization	O(N) expected with canonical keys; $O(\sum_{i}b_{i}^{2})$ inside fallback buckets	*N* is raw claim records and $b_{i}$ are bucket sizes for claims that require additional similarity checks.
Homotopy localization	O(N) expected grouping plus support aggregation	Implemented primarily as canonical-key grouping in Csql; expensive comparisons are confined to candidate buckets.
Regime gluing	O(UR+E) grouping and projection	*U* is localized classes, *R* regime or polarity labels, and *E* stored support edges/provenance links.
Manifold and neighborhood diagnostics	$O(N^{2}d)$ for exact pairwise neighborhoods; lower with approximate neighbors	*d* is embedding dimension; saved runs bound rendering through topk, radii, and maxnodes.
Observed compatibility summaries	$O(\sum_{j}v_{j}^{3})$ worst case per localized family	$v_{j}$ is the number of vertices in a family; practical runs cap local model size and report candidate-triangle closure diagnostics.
Artifact rendering and SQL summaries	O(NlogN+A)	Group-by/index operations dominate Csql summaries; *A* is the number of rendered artifacts, figures, and dashboard sections.

## Appendix B. Reproducing the Results with the GitHub Cliff Implementation

The experiments described in this paper can be reproduced from the public Cliff implementation, which is organized in the companion GitHub repository CLIFF_CatAgi, version c060d94759d01f0bf3471d85d4c6e1ad4e9589a5 (1 May 2026), (https://github.com/sridharmahadevan/CLIFF_CatAgi (accessed on 10 April 2026)). That repository provides the natural-language interface, route-selection logic, dependency-resolution layer, and saved-artifact surface used to launch Democritus runs from ordinary text queries. In the terminology of the public release, Cliff is the *Consciousness Interface to Functor Flow*: a user-facing layer that routes a request into deeper workflows and then promotes the resulting synthesis artifact back into a bounded dashboard or report.

**Table A3:** Artifact and reproducibility checklist for the revised experiments. The public compact bundles are meant for inspection and smoke reproduction; the full archived runs contain the heavier database and sweep artifacts used for the numerical summaries.

Reproducibility Item	Where It Appears	Purpose
Public implementation	CLIFF_CatAgi, especially the functorflow_v3 package	Route selection, document acquisition, batch execution, corpus synthesis, and public artifact export.
Normalization helpers	causal_homotopy.py	Canonical phrase and relation normalization used by localization and regime views.
Csql bundle builder	csql_bundle.py	SQLite schema, claims table, homotopy-localized claim view, and regime-gluing surface view.
Corpus synthesis	democritus_corpus_synthesis.py	Loads Csql views, computes homotopy and regime summaries, and renders dashboards.
Public compact examples	Two bundles under examples/democritus/	GitHub-friendly bundles with query plans, selected documents, stage timings, Markdown summaries, and manifold images.
Heavyweight archived runs	Local saved Cliff/Democritus run directories referenced in the case studies	Preserve full PDFs, sweep outputs, SQLite bundles, dashboards, LCM galleries, and richer metadata omitted from compact public exports.
Stage timing summaries	batch_stage_summary.json	Records completed stages, document counts, errors, and elapsed time by agent.
Frozen document covers	Selected-document, example-manifest, or acquired-corpus manifest JSON	Allows reruns against a fixed cover rather than relying on live retrieval.
Frozen extraction benchmark	Public benchmark directory	Contains the harness, tests, manifest, predictions, metrics, prompts, model identifiers, and hashes for Table 8; the externally obtained AltLex data are not redistributed.

The external extraction comparison is independently reproducible from the public benchmark directory experiments/causal_extraction_benchmark/. The frozen grouped CSV has SHA-256 093ae83baf64339203130928b810e7208895335148cfef403a051195a2dee5ce. The directory records the 404 example identifiers, model names, exact prompt, raw model responses, validated spans, prediction hashes, and metric JSON. The dataset itself is not redistributed because the UniCausal repository notes unresolved licensing information for the underlying AltLex resource. Four unit tests cover tagged-span reconstruction, verbatim validation, scoring, and multi-relation parsing.

The lightweight installation path is:

cd CLIFF_CatAgi
python3 -m venv .venv
source .venv/bin/activate
pip install -r requirements.txt
pip install -e .

For the Democritus route used in this paper, one also needs access to the companion Democritus repository and its model/runtime dependencies. The current Cliff resolver looks for such dependencies either under third_party/ or as sibling repositories in a shared workspace, and also supports environment-variable overrides such as Cliff_DEMOCRITUS_ROOT, Cliff_COURSE_REPO_ROOT, and Cliff_BOOK_PDF_PATH. A typical workspace layout is therefore:

 workspace/
   CLIFF_CatAgi/
   Democritus_OpenAI/
   Category-Theory-for-AGI-UMass-CMPSCI-692CT/

**Table A4:** Experimental settings exposed by the public Cliff/Democritus route. These settings are recorded in run summaries and can be overridden from the command line.

Setting	Default or Value Used in Reported Runs	Reproducibility Note
Execution mode	deep for full runs; quick or interactive for inspection	Controls whether the route stops early or completes corpus synthesis.
Document count	Target-docs flag set to 5 for the ocean example; direct PDF input for the penguin run	Fixed manifests or direct PDFs are preferred for paper reproduction.
Retrieval backend	Automatic routing, with manifest, direct-file, and direct-directory modes available	Live retrieval can drift; archived selected-document manifests freeze the cover.
Root-topic strategy	summary_guided	Used to recover document-local topic frontiers before claim extraction.
Topic and statement caps	Depth 3; at most 100 topics; 2 statements per causal question	These bound the topic expansion and LLM extraction workload.
Statement generation	Batch size 16; maximum 192 tokens per statement	Controls batching and maximum generation length for candidate claims.
Manifold settings	manifold-mode = full, topk = 200, radii = 1,2,3, maxnodes = 10,20,30,40,60	Determines the neighborhood and local-causal-model sweeps used in figures and diagnostics.
Claim display and tiers	topk-models = 5, topk-claims = 30, tier1 = 0.60, tier2 = 0.30	Controls report truncation and tier labels; underlying Csql records remain available.
Rendering	top-*k* PNG rendering on; png-dpi = 200	Generates the local graph images and manifold figures used in archived reports.

Once the environment is available, a one-shot corpus run can be launched directly from the Cliff entrypoint. For example, a five-document ocean-temperature synthesis can be invoked as:

  python3 -m functorflow_v3.cliff \
   --query "Analyze 5 recent studies on rising ocean temperatures" \
   --route democritus \
   --execution-mode deep \
   --outdir /tmp/cliff-ocean \
   --democritus-target-docs 5

If a fixed local corpus is desired instead of live retrieval, Cliff also exposes manifest- and file-based inputs. In particular, –democritus-manifest, –democritus-input-pdf, and –democritus-input-pdf-dir are often preferable for paper reproduction because they freeze the cover explicitly. The same entrypoint can therefore be used either for retrieval-driven runs or for locked manifest-based reruns of previously selected documents.

An older companion branch, TCC-enabled CLIFF, also included a specialized tcc_atlas route for querying the Testing Causal Claims economics atlas directly through its curated atlas/Csql representation, without live document retrieval. That branch is not the main implementation analyzed in this paper, but it is a useful proof-of-concept that the Cliff interface can sit on top of substantially larger precompiled claim repositories as well as the document-level Democritus workflows emphasized here.

The output layout is designed to preserve the exact artifacts discussed in the paper. Each routed Cliff session writes a route-specific directory under the chosen outdir; for the present experiments, the relevant subtree is outdir/democritus/. That directory contains a run summary, the archived Democritus batch directory, and the generated corpus-synthesis dashboard. In the current implementation, the synthesized HTML artifact is written under the democritus_runs/corpus_synthesis/ subtree, while the route summary records the paths to the corresponding Csql bundle and batch outputs. Those files are precisely the bridge between the theoretical constructions described in the body of the paper and the reproducible computational objects used in the experiments.

For practical verification, the public repository already includes regression tests for the Cliff router and major Democritus-facing surfaces. A minimal smoke-test pass is:

python3 -m unittest tests.test_query_router_agentic tests.test_cliff

and broader local verification can be obtained with python3 -m unittest. In this sense, the manuscript’s experimental layer is not only conceptually reproducible but also tied to an executable public interface that exposes routing, artifact production, and dependency resolution in a form that other researchers can inspect directly.

## Appendix C. Archived Source Covers and Manifold Diagnostics

To keep the experiments auditable, Table A5 records the source texts preserved in the surviving saved runs used for the main case studies. The red-wine and ocean runs retain exact document identities. The Mediterranean archive survives only as an interactive checkpoint with four selected texts out of twelve discovered candidates rather than as a frozen final ten-document manifest, while the emperor-penguin archive preserves the single acquired PDF identity but not richer publisher metadata.

**Table A5:** Source-document provenance recovered from the surviving archived Cliff/Democritus runs used in the paper.

Case Study	Archived Source Texts	Archive Note
Mediterranean diet	*Multi-Omic Insights Into Mediterranean Diet-Associated Microbiota* (https://pmc.ncbi.nlm.nih.gov/articles/PMC13049501/ (accessed on 10 April 2026)); *Epigenetics of Mediterranean Diet: Altering Disease Risk* (https://doi.org/10.1007/978-3-319-27969-5_15); *Cancer and the Mediterranean Diet* (https://doi.org/10.1201/9781420042221-15); *Mediterranean Diet and Longevity* (https://doi.org/10.1201/9781420042221-12).	Interactive checkpoint preserved four selected texts from twelve discovered candidates.
Red wine	*Red Wine Consumption and Cardiovascular Health* (https://pmc.ncbi.nlm.nih.gov/articles/PMC6804046/ (accessed on 10 April 2026)); *Resveratrol: A Double-Edged Sword in Health Benefits* (https://pmc.ncbi.nlm.nih.gov/articles/PMC6164842/ (accessed on 10 April 2026)).	Exact paired inputs used for the pullback/pushout collation experiment.
Emperor penguins	Archived Washington Post emperor-penguin PDF from “Emperor penguins have just been declared endangered” (https://www.washingtonpost.com/climate-environment/2026/04/09/emperor-penguin-endangered-antarctic-fur-seal/ (accessed on 10 April 2026)).	Single-document run preserves the PDF identity but not full article metadata.
Rising ocean temperatures	*Pre-requisite conditions for the intensification of pre-monsoon cyclones over the Bay of Bengal* (https://pmc.ncbi.nlm.nih.gov/articles/PMC12474882/ (accessed on 10 April 2026)); *A standardized indicator reveals sharper increases in heat related mortality in temperate zone cities worldwide* (https://pmc.ncbi.nlm.nih.gov/articles/PMC12769731/ (accessed on 10 April 2026)); *A universal concept for melting in mantle upwellings* (https://pmc.ncbi.nlm.nih.gov/articles/PMC12935539/ (accessed on 10 April 2026)); *Major heat wave in the North Atlantic had widespread and lasting impacts on marine life* (https://pmc.ncbi.nlm.nih.gov/articles/PMC12757028/ (accessed on 10 April 2026)); *Modeling the metabolic evolution of mixotrophic phytoplankton in response to rising ocean surface temperatures* (https://doi.org/10.1186/s12862-022-02092-9); *Rising temperatures in the subtropical North Atlantic Ocean over the past 35 years* (https://doi.org/10.1038/369048a0).	Query asked for five studies, but the preserved cover contains six acquired documents spanning neighboring subfields.

## Appendix D. Additional Categorical Background

The homotopy construction used here sits inside a broader categorical setting. The body of the paper proves the elementary relative-category and localization properties needed by the implementation, formulates homotopical repair of a causal-discourse learning sketch, and gives a conditional bridge into causal-model equivalence. Many directions remain outside the present scope: a tangent lift that would turn base repair into infinitesimal non-compositionality, Markov-category semantics, richer topos-internal intervention logics, derived Kan extensions, and a model- or (∞,1)-categorical completion of the observed compatibility complexes. For the present paper, the computational core remains localization of causal mentions at normalization-induced weak equivalences followed by finite, provenance-preserving compatibility diagnostics.

The public interface implementation lives in Cliff_CatAgi: within functorflow_v3/, modules such as textbook_backstop.py and democritus_corpus_synthesis.py expose the route orchestration and corpus-synthesis layer used by the present system. The aim of the present paper is therefore focused: here we study the homotopy-localization and causal-gluing behavior of the system, while leaving the fuller learning machinery outside the paper’s evidential scope.

## Appendix E. Additional Experimental Directions

The saved runs also suggest several directions that are not yet fully developed in the current manuscript. Broad queries such as climate change and economic inflation show that cover selection and topic partitioning are prior problems for any downstream gluing analysis. Single-document runs show that many remaining disconnected families are really clause-tail segmentation issues. Large archived corpora also open the possibility of comparative studies across versions of Democritus, using the same saved runs to measure how topic drift, homotopy localization, and regime gluing changed over time.
